# China’s criminal incidental civil public interest litigation in food safety: practice review and pathways to improvement

**DOI:** 10.3389/fnut.2026.1852511

**Published:** 2026-06-10

**Authors:** Xiao Han, Bingxiang Li

**Affiliations:** Department of International Law, China Foreign Affairs University, Beijing, China

**Keywords:** Chinese procuratorial public interest litigation, criminal precedence, food safety, plea leniency, punitive damages

## Abstract

**Introduction:**

Criminal deterrence and the restoration of public interests are integrated in the system of criminal incidental civil public interest litigation in the field of food safety. However, the inherent tension between criminal and civil law within this framework has created practical challenges.

**Methods:**

Drawing on an empirical analysis of 677 judicial judgments, this study identifies several systemic impediments to effective judicial decision-making.

**Results:**

First, an ambiguous definition of “social public interest” makes it difficult to initiate litigation and leads to the arbitrary extension of the case scope. Second, public interest repair is delayed by the rigid adherence to the principle of “criminal liability first, and then civil liability.” Third, uncertainty regarding the applicable standards of proof arises because civil adjudication heavily relies on criminal proceedings. Furthermore, judicial discretion is unchecked in the application of punitive damages, judgments are not effectively enforced, and regulatory oversight is inadequate.

**Discussion:**

In light of the forthcoming Public Interest Litigation Law of the People’s Republic of China, it is imperative to establish a flexible trial model oriented toward repair, to clarify the distinctions between criminal and civil standards of proof, and to further refine the rules governing the discretion and administration of punitive damages. The efficiency and effectiveness of this system can be enhanced by strengthening the procedural framework for criminal incidental civil public interest litigation, thereby contributing to comprehensive governance that integrates punishment, prevention, and repair in safeguarding food safety.

## 1 Introduction

When a People’s Procuratorate initiates a criminal public prosecution for criminal acts in the field of food and drug safety that infringe upon the lawful rights and interests of numerous consumers or harm the social public interest, it may concurrently file a civil public interest litigation incidental to criminal proceedings with the People’s Court,” according to Article 20 of the newly revised Interpretation of the Supreme People’s Court and the Supreme People’s Procuratorate on Several Issues Concerning the Application of Law to Public Interest Lawsuits Brought by the People’s Procuratorates (2020). By balancing private and public interests, the criminal incidental civil public interest litigation system in the area of food safety represents an important turning point in the development of China’s public interest litigation system, as it fuses procedural innovation with practical sensitivity. According to the *White Paper on Procuratorial Public Interest Litigation (2025)* released by the Supreme People’s Procuratorate in March 2026, in 2025, procuratorial authorities nationwide handled a total of 136,000 public interest litigation cases, including 12,000 civil public interest litigation cases. A total of 7,305 public interest litigation cases were brought before the courts, with 99.8% receiving judicial support, and the number of cases in the field of food and drug safety continued to rise.

At the level of legal regulation, since the Fourth Plenary Session of the 18th CPC Central Committee explicitly proposed the strategic deployment to “explore the establishment of a system for procuratorial public interest litigation” (1) in 2014, the system of procuratorial public interest litigation has evolved from nothing to a fully-fledged framework, following a logical trajectory of “policy promotion—legislative confirmation—normative refinement”.^1^ In October 2025, the recommendations for the 15th Five-Year Plan, reviewed and adopted at the Fourth Plenary Session of the 20th CPC Central Committee, called for “strengthening procuratorial oversight and enhancing public interest litigation”. (2) Shortly thereafter, the Draft Law on Public Interest Litigation by Procuratorial Organs (hereinafter referred to as the “Draft”) was submitted for deliberation at the 18th Session of the 14th National People’s Congress Standing Committee. This marked the formal entry of public interest litigation by procuratorial organs into the realm of specialized legislation, following more than a decade of practical exploration. The institutional framework was advancing toward greater rule of law, refinement, and comprehensiveness. At the same time, the Food Safety Law was most recently amended in September 2025, further strengthening the legal liability system in the field of food safety. Together with the Consumer Rights Protection Law, it provides a clearer and more robust legal basis for claims of punitive damages in criminal incidental civil public interest litigation, thereby injecting new substantive legal momentum into the operation of the procuratorial public interest litigation system. Globally, judicial approaches to public interest protection take diverse institutional forms. The privately enforced class actions in the United States, the representative actions brought by qualified entities in the European Union, India’s epistolary jurisdiction that proactively relaxes standing requirements through judicial activism, and Brazil’s public civil action (*Ação Civil Pública*) statutorily authorizing independent enforcement by the procuratorate—each of these demonstrates the institutional possibilities for public interest relief from different perspectives. China’s distinctive model, in which the procuratorate takes the lead and resolves civil disputes incidentally within criminal proceedings, differs substantially from the typical approaches of the aforementioned jurisdictions and warrants comprehensive examination from both theoretical and practical dimensions.

At the theoretical level, food safety is closely tied to the health and safety of the general public (3–5); consequently, both academic circles and practical departments have devoted significant attention to the relevant theories and institutional norms governing public interest litigation in food safety cases. In the international academic community, Wang (6) coined the concept of “judiciary-led public interest litigation”. From a comparative law perspective, he examined the Chinese model alongside those of India, Brazil, and other countries, arguing that the institutional characteristic of this system lies in the “supervision-cooperation” dual relationship between courts and procuratorial authorities. Qi and Yu (7), on the other hand, defined procuratorial public interest litigation as an institutional tool of “responsive governance” from the perspective of governance functions. Ding (8), drawing on comparative law and sociological perspectives, systematically analyzed the institutional characteristics of administrative public interest litigation initiated by procuratorial authorities and its impact on the judicialization of administrative governance.

Within the domestic academic community, Liu Yi systematically examined the institutional logic and development trajectory of procuratorial public interest litigation from the dimensions of state governance and empirical testing (9, 10), while Zhang (11) adopted a legal interest analysis approach to conduct in-depth research on the composition of legal interests and their positioning in food safety public interest litigation. Regarding the punitive damages system, Yang Huixin et al. conducted a theoretical analysis focusing on the systemic positioning and legal policy calibration of punitive damagest (12, 13). Su (14) offered valuable insights on the legitimacy and practical application of punitive damages in the food and drug safety sector. Wang (15) explored the specific application of punitive damages in criminal incidental civil public interest litigation from the perspectives of civil application and criminal coordination. Ren and Fang (16) further analyzed the application dilemmas and resolution pathways for punitive damages in food safety civil public interest litigation, while Nie and Shen (17) proposed institutional concepts for punitive damages in food and drug safety civil public interest litigation from a legislative perspective. Regarding the procedural structure of criminal incidental civil public interest litigation, Xie and Guo (18) revealed the procedural dependency of such litigation on criminal proceedings, while Cheng (19) took a negative stance toward the system, and Xiao (20) defended it from a functional and value-based perspective. In terms of empirical research, Long et al. conducted an empirical review of the set-off between compensation and fines and the application of relevant laws (21, 22).

Overall, existing research has either focused on the doctrinal interpretation of institutional positioning or concentrated on the normative analysis of a single procedural stage. Systematic and comprehensive examination of the practical patterns of criminal incidental civil public interest litigation in food safety from an integrated perspective remains scarce. Accordingly, this paper compiles publicly available judicial rulings and utilizes empirical statistical data to outline the practical landscape of criminal incidental civil public interest litigation in the food safety sector over the past 5 years, integrating and analyzing the systemic bottlenecks within this framework. The study aims to make marginal contributions at three levels. At the theoretical level, it proposes and substantiates the analytical concept of “behavior-centric approach,” providing a theoretical tool to understand the arbitrary expansion of the scope of public interest litigation; at the same time, using empirical data, it tests the application effect of the “criminal priority” principle in food safety public interest litigation, finding that its legitimacy—rooted in private interest litigation—is difficult to sustain in the context of mass torts. At the methodological level, based on a coded analysis of 677 recent judicial judgments, it constructs a five-stage analytical framework encompassing “pre-litigation initiation—case adjudication—criminal-civil proof—application of punitive damages—judgment enforcement,” thereby overcoming the fragmentation of previous studies that often focused on only a single stage. On the core issue, building on the theoretical debate between the “derivative right of claim in public interest litigation” and the “statutory private right of claim,” it proposes that future legislation should create an independent “public interest penalty” right of claim, so that the nature of punitive damages in public interest litigation is completely distinguished from private interest compensation.

## 2 Materials and methods

### 2.1 Research design

This study employed a quantitative content analysis approach to systematically examine the operational status and structural dilemmas of criminal incidental civil public interest litigation in food safety. A structured coding framework was developed to extract key variables from each judicial document, covering case characteristics, procedural arrangements, substantive rulings, and enforcement outcomes.

### 2.2 Data sources and sampling

Judicial documents were retrieved from two publicly available databases: China Judgments Online and the Peking University Law Database. The search used the keyword “criminal incidental civil public interest litigation,” restricted the cause of action to “Crime of Producing and Selling Counterfeit or Substandard Goods,” and limited the date range from January 1, 2021, to November 30, 2025. A total of 949 documents were initially retrieved.

Through manual screening, procedural documents and blank judgments were first excluded. Documents unrelated to food safety or ultimately classified as drug-related crimes were further removed, and duplicate cases were deduplicated. This process yielded 677 valid samples, including 671 first-instance documents (660 judgments and 11 mediation agreements), 4 s-instance judgments, and 2 retrial judgments. A detailed summary of the 677 cases is provided in Supplementary Table S1.

### 2.3 Variable definition and classification

Based on the full litigation life-cycle perspective of initiation, trial, adjudication, and enforcement, this study is organized into four dimensions: case characteristics, procedural features, adjudicatory outcomes, and liability assumption and enforcement. The variables under each dimension correspond to the core points of legal-institutional concern that arise during the operation of the criminal incidental civil public interest litigation system in the field of food safety. The definition of each variable, the method by which it is identified in judicial documents, and an illustrative example are provided in Supplementary Table S1. The meaning of each variable and the reasons for its selection are explained below.

#### 2.3.1 Case characteristics dimension: identifying enforcement priorities and risk distribution

The case characteristics dimension includes two variables: cause of action and type of problematic food.

The cause of action refers to the criminal charge stated in the judgment. The reason for selecting this variable is that the cause of action reflects the legal basis for criminal prosecution. By analyzing the distribution of different charges, it is possible to identify which offenses are the current focus of enforcement in food safety crimes, and further to examine how the priorities of criminal prosecution affect the adjudication of civil public interest claims.

Types of problematic food are classified according to the function of the food or its main hazard characteristics. The reason for selecting this variable is that food safety risks take specific forms in judicial practice. This variable allows those forms to be categorized and presented, thereby providing an empirical basis for subsequent discussion of which risk areas should be the main focus of judicial regulation.

#### 2.3.2 Procedural features dimension: three key nodes for examining procedural propriety

The procedural features dimension includes three variables: implementation status of the pre-litigation public announcement, trial sequence, and admissibility of expert assessment reports.

Implementation status of the pre-litigation public announcement examines whether the judgment records the performance of the announcement procedure and the type of medium used. This variable was selected because the pre-litigation public announcement is a statutory prerequisite for procuratorial public interest litigation, designed to safeguard the right of action of other eligible entities. By observing the implementation status and choice of medium, it is possible to assess whether the pre-litigation announcement has become a mere formality or whether it has substantially restricted public awareness and participation.

The trial sequence focuses on the chronological order in which the civil public interest litigation portion and the criminal portion are heard. This variable was selected because the rigid application of the principle “criminal proceedings first, civil proceedings second” is a core theoretical issue of this study. By observing the distribution of trial sequences, we can directly assess whether civil remedies are placed in a subordinate and delayed position in the procedural timeline.

The admissibility of expert assessment reports examines whether specialized evidence, such as food safety risk assessments, is admitted in litigation. This variable was selected because such evidence is subject to a more lenient standard of review under civil evidentiary rules, but in criminal incidental civil proceedings, it is often excluded due to the spillover effect of criminal evidentiary standards. This variable directly tests whether the conflation of criminal and civil standards of proof has narrowed the scope for proving public interest harm.

#### 2.3.3 Adjudicatory outcomes dimension: measuring judicial discretion and the balance between criminal and civil sanctions

The adjudicatory outcomes dimension includes four variables: the multiplier of punitive damages, the amount of punitive damages, the amount of criminal fines, and the ratio of fines to punitive damages (F/P ratio).

The multiplier of punitive damages refers to the multiplier ultimately supported by the court. The reason for selecting this variable is that the multiplier directly reflects the severity of the court’s sanction against food safety violations, and its distribution can reveal whether judicial discretion is exercised consistently.

The amount of punitive damages refers to the total amount awarded by the court in a given case. The reason for selecting this variable is that the degree of variation in the amounts across cases can indicate whether the outcomes of judicial discretion are significantly dispersed.

The amount of criminal fines refers to the total fine imposed in a given case. The reason for selecting this variable is that the fine reflects the intensity of the criminal sanction and serves as a necessary reference for assessing the coordination between criminal and civil property sanctions.

The ratio of fines to punitive damages (F/P ratio) is calculated as the criminal fine divided by the amount of punitive damages. The reason for selecting this variable is that this ratio directly reflects the relative weight between criminal sanctions and civil public interest remedies. The distribution of the ratio can reveal whether the courts have achieved a stable balance between the two types of sanctions.

#### 2.3.4 Liability and enforcement dimension: tracking the final effectiveness of judgments

The liability and enforcement dimension includes three variables: the imposition of probation, the imposition of occupational bans, and the allocation of punitive damages.

The imposition of probation concerns whether the defendant is sentenced to probation. This variable was selected because the application of probation reflects the extent to which courts exercise leniency in the actual execution of sentences against food safety offenders, and it directly affects whether the deterrent effect of criminal punishment is effectively achieved in practice.

The imposition of occupational bans concerns whether the court simultaneously issues a prohibition order. This variable was selected because occupational bans are an important preventive judicial measure to prevent offenders from re-entering the food industry, and their application reveals the actual extent to which preventive justice is used in the field of food crimes.

The allocation of punitive damages concerns whether the judicial document explicitly specifies the managing entity and the distribution channel for the compensatory funds. This variable was selected because the unclear destination of punitive damages is a systemic flaw repeatedly discussed in academic literature. By examining how judgments address this issue, we can directly reveal whether there is an institutional gap in translating public interest remediation from judicial decisions into reality.

#### 2.3.5 Summary table of variables

For ease of reference, the definitions and classification rules for all the above variables are summarised below (see Table 1).

**Table 1:** Variable definitions and classification.

Variable category	Variable name	Definition and classification rules
Case characteristics	Cause of action	Criminal charge stated in the judgment, including: crime of producing or selling toxic or harmful food; crime of producing or selling food that does not meet safety standards; crime of producing or selling counterfeit or substandard products; crime of selling goods bearing counterfeit registered trademarks; crime of counterfeiting registered trademarks; and other types
	Type of problematic food	Specific category of food involved in the case, including: fake health products; weight-loss products; alum-containing fried dough sticks and other flour products; problematic meat products; seasonings containing poppy shells; bean sprouts with illegal additives; “gutter oil” hotpot; vegetables with excessive pesticide residues; marinated products with excessive nitrite levels; health products containing pharmaceutical ingredients; and other types
Procedural features	Implementation status of pre-litigation announcement	Whether the judgment records the implementation of the pre-litigation announcement procedure, including: performed and the publication platform specified; performed but the platform not specified; and not mentioned
	Pre-litigation announcement platform	Media channel through which the pre-litigation announcement was published, including: internal procuratorial platforms (Justice Net, Procuratorial Daily, etc.); public media (People’s Court Daily, etc.); and other or unidentifiable channels
	Trial sequence	Temporal order between the adjudication of criminal and civil liability, including: criminal proceedings first, then civil proceedings; parallel or other flexible arrangements; and not specified
	Admissibility of expert assessment reports	Whether professional evidence such as food safety risk assessment reports and expert opinions is admitted, including: admitted under criminal evidentiary standards; excluded as “speculative” evidence; and not involved
Punitive damages	Multiplier of punitive damages	The multiplier applied by the court in awarding punitive damages, including: threefold compensation; tenfold compensation; statutory minimum of 1,000 yuan; and other multiplier as determined by the court
	Amount of punitive damages	The amount of punitive damages stated in the judgment (in yuan). Grouped by range: below 1,000 yuan; 1,000 to below 10,000 yuan; 10,000 to below 100,000 yuan; 100,000 to below 500,000 yuan; 500,000 to below 1,000,000 yuan; and 1,000,000 yuan or above
	Amount of criminal fine	The amount of criminal fine stated in the judgment (in yuan), used to measure the intensity of property sanctions on the criminal side
	Ratio of fine to punitive damages (F/P ratio)	Calculated as total criminal fine divided by total punitive damages, used to measure the structural relationship between criminal and civil property sanctions
Liability and enforcement	Types of non-monetary remedies	Non-property remedies ordered by the court, including: public apology only; product recall, destruction or warning only; multiple remedies combined; and no non-monetary remedy applied
	Imposition of probation	Whether the defendant was granted probation, including: probation granted; and not granted
	Imposition of occupational ban	Whether the court issued a prohibition order, including: occupational ban imposed; and not imposed
	Allocation of punitive damages	The recipient or managing entity of the awarded funds as stated in the judgment, including: remitted to local treasury; paid to procuratorial authorities; transferred to a public interest fund or special account; and not specified

### 2.4 Figures

**Figure 1:**
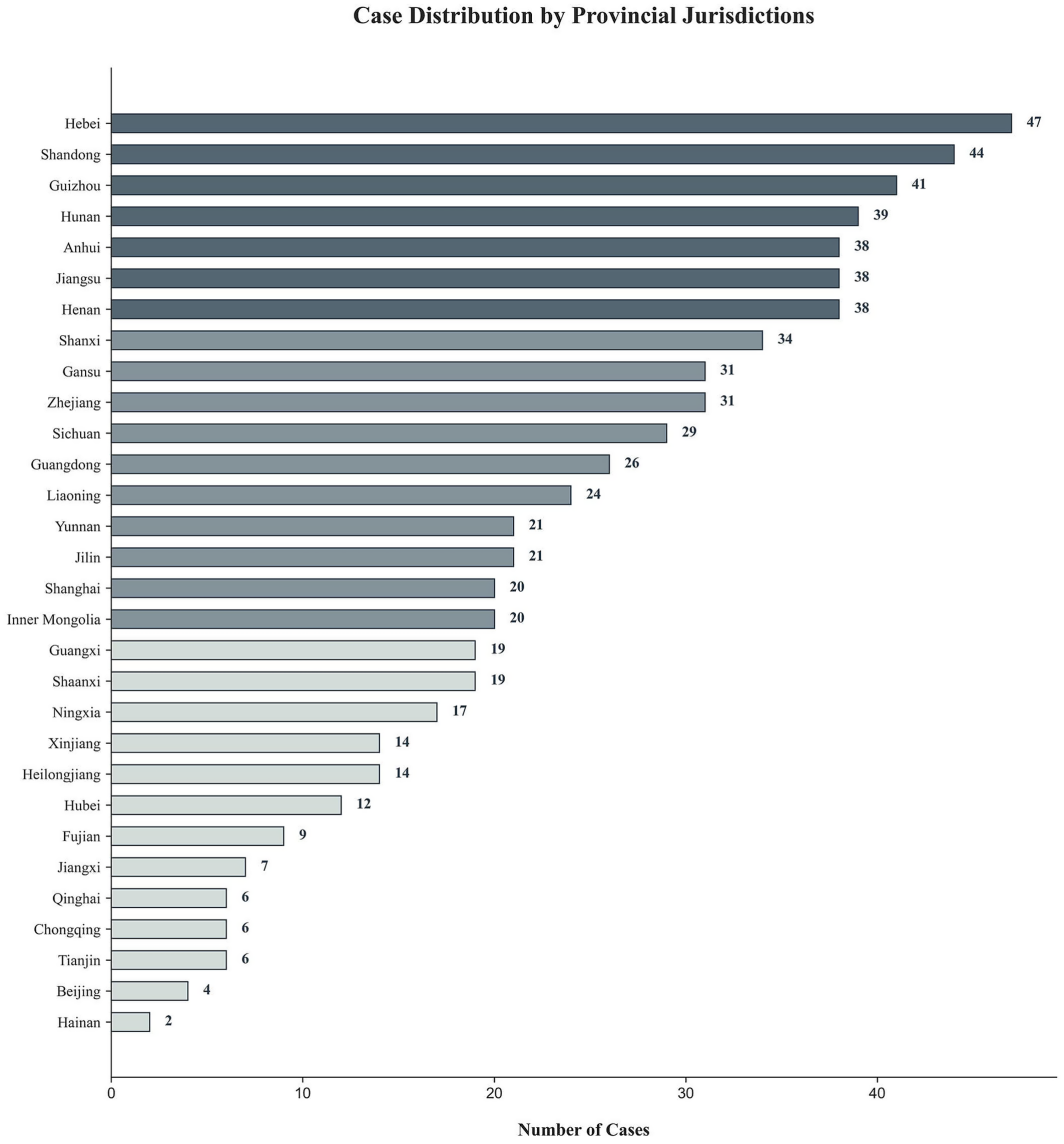
Geographical distribution of 677 food safety cases across 30 provincial-level administrative regions in China (2021–2025). The map shows that cases are concentrated in North China, East China, and Southwest China, with Hebei, Shandong, and Guizhou provinces having the highest numbers.

**Figure 2:**
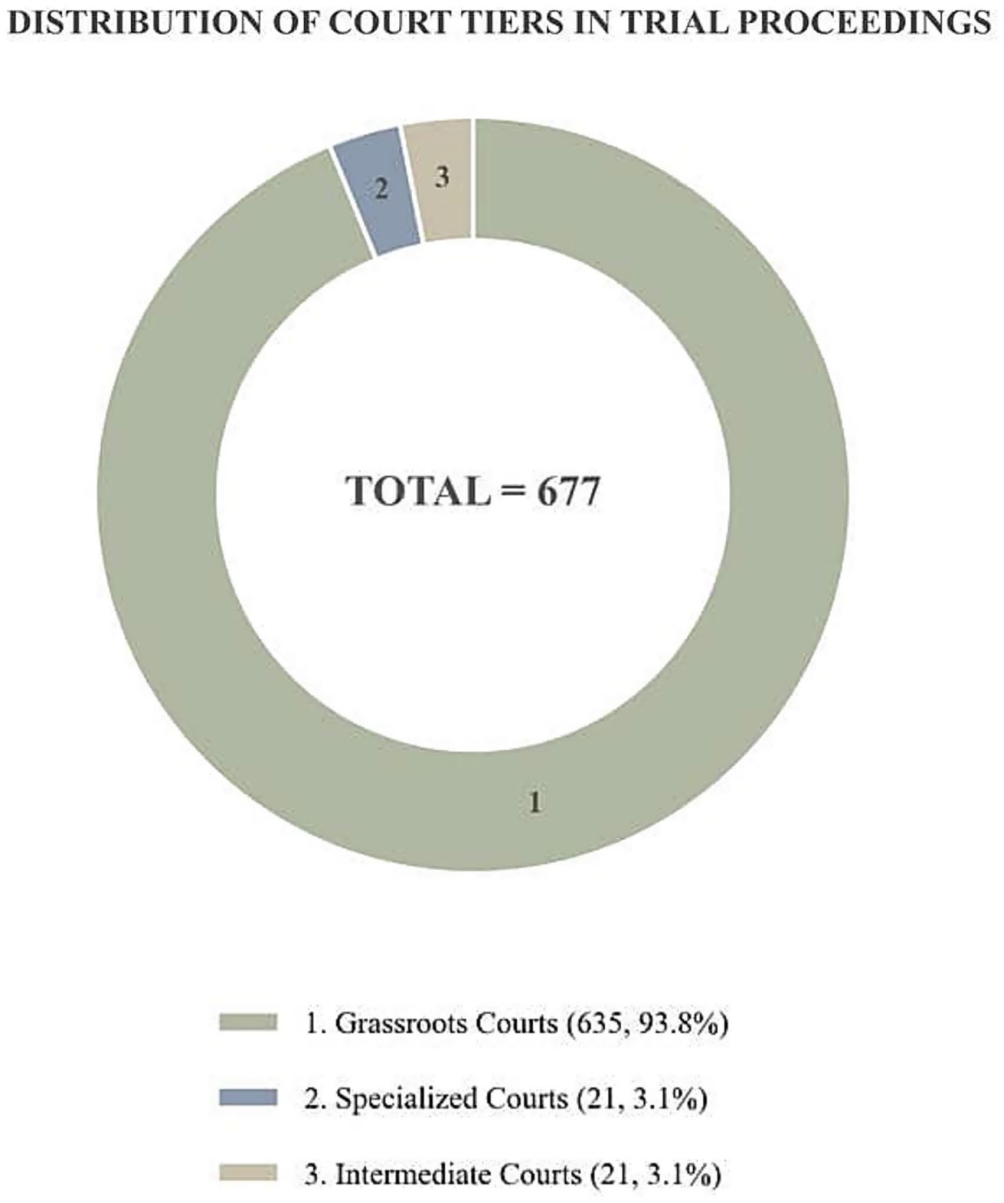
Distribution of trial levels: first-instance, second-instance, and retrial cases. Over 93% of cases were adjudicated by grassroots courts, forming a distinctive “decentralized” adjudication structure.

**Figure 3:**
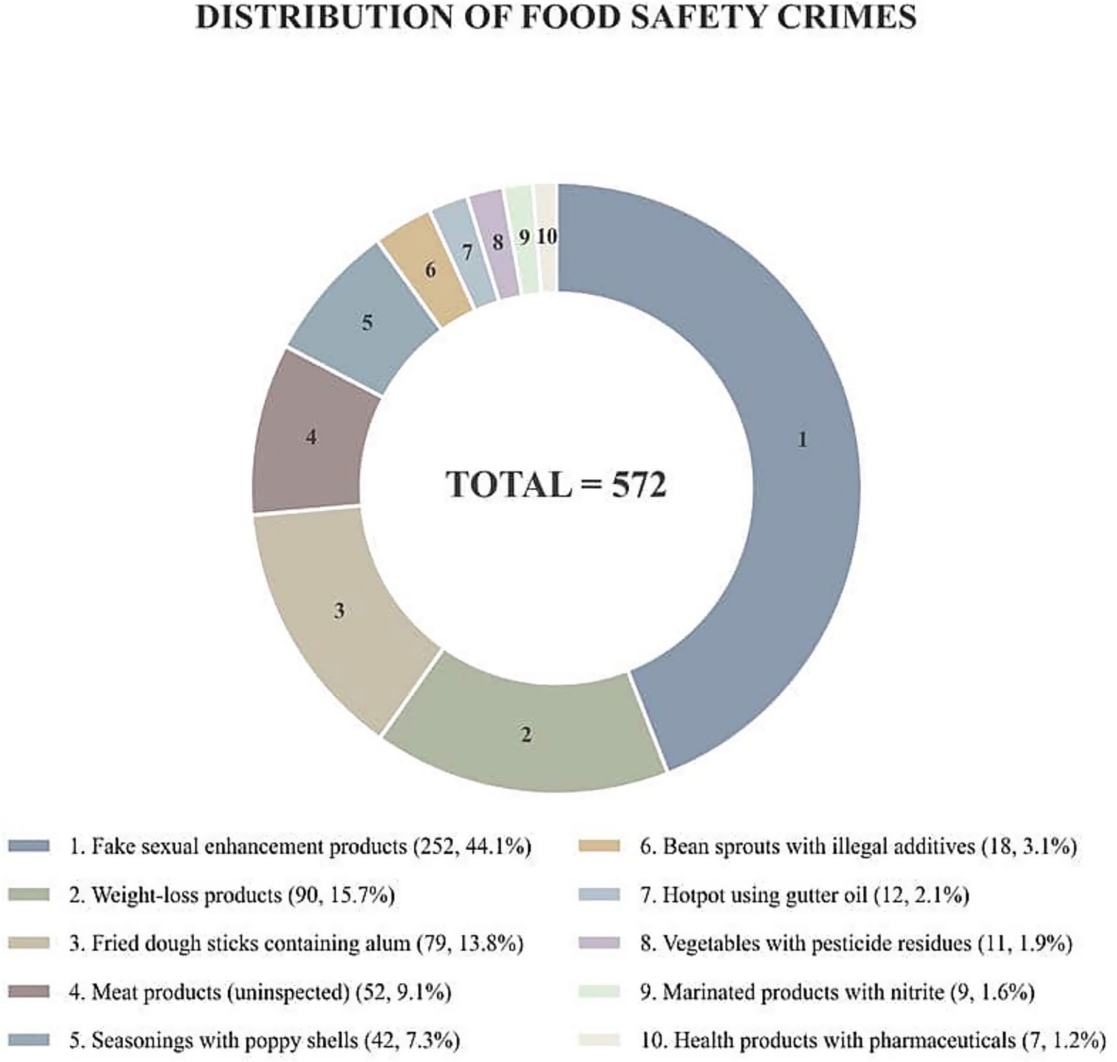
Types of problematic food identified in the sample cases. The top five categories—fake sexual enhancement products, weight-loss products, fried dough sticks containing alum, problematic meat products, and seasonings containing poppy shells—account for over 75% of total case occurrences.

**Figure 4:**
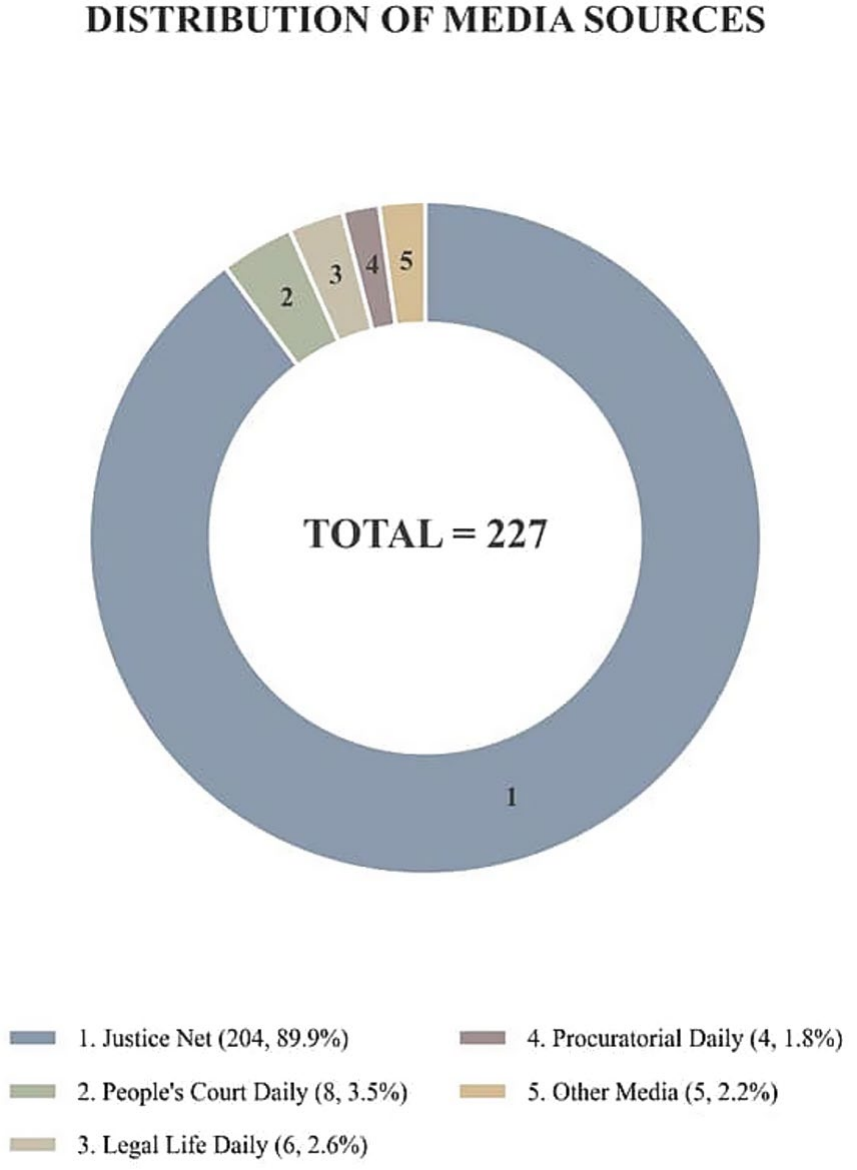
Distribution of pre-litigation announcement websites used by procuratorial authorities. Among the 227 cases where the announcement medium was specified, 91.7% (208 cases) published notices on internal platforms (justice net and the procuratorial Daily), while the People’s Court Daily and other public media accounted for only 8.3%.

**Figure 5:**
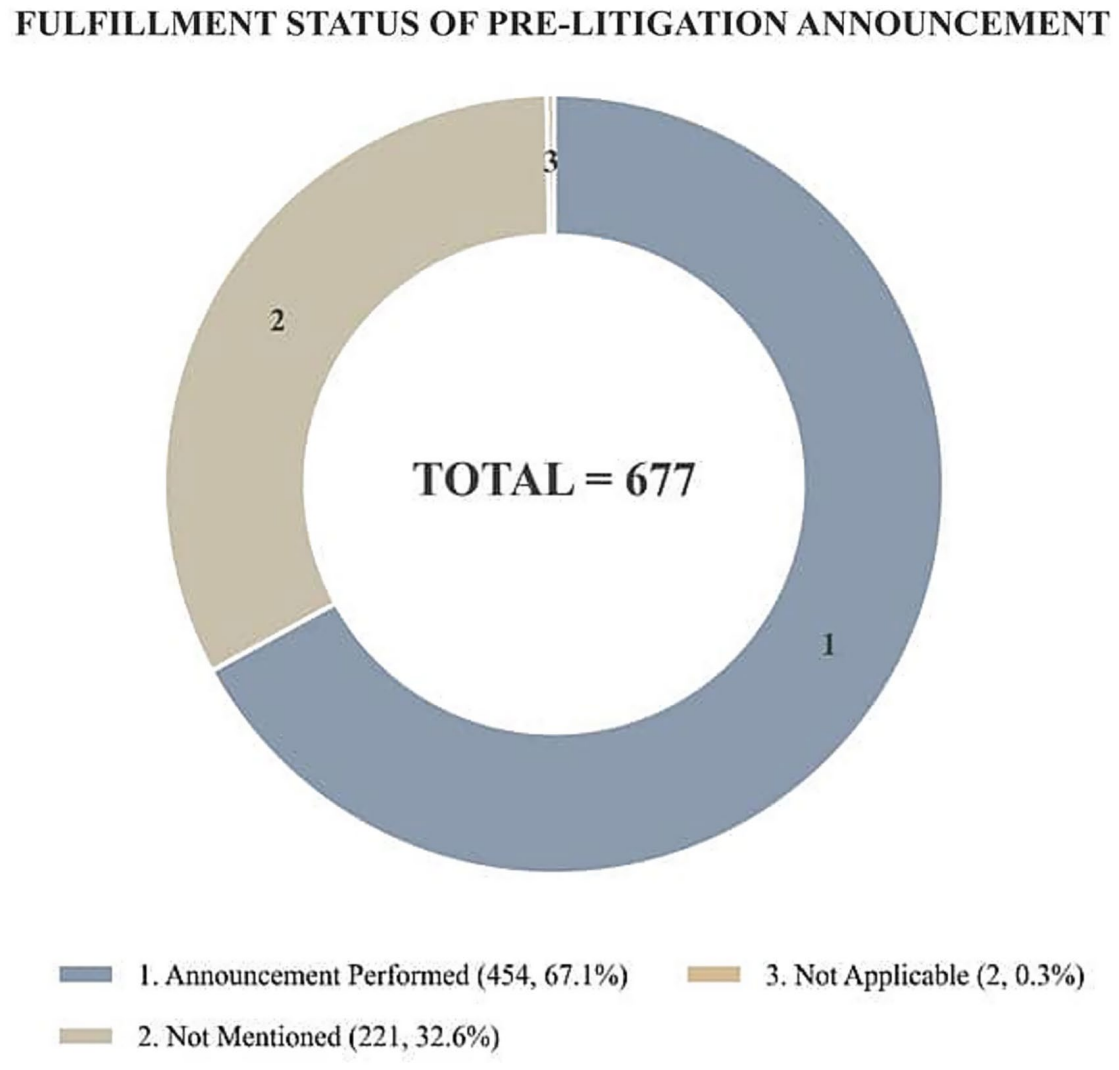
Implementation of pre-litigation announcements in the sample cases. Among 677 cases, 32.6% (221 cases) made no mention of the procedure; among the 454 cases where the procedure was performed, only half (227 cases) specified the announcement medium in the court judgment.

**Figure 6:**
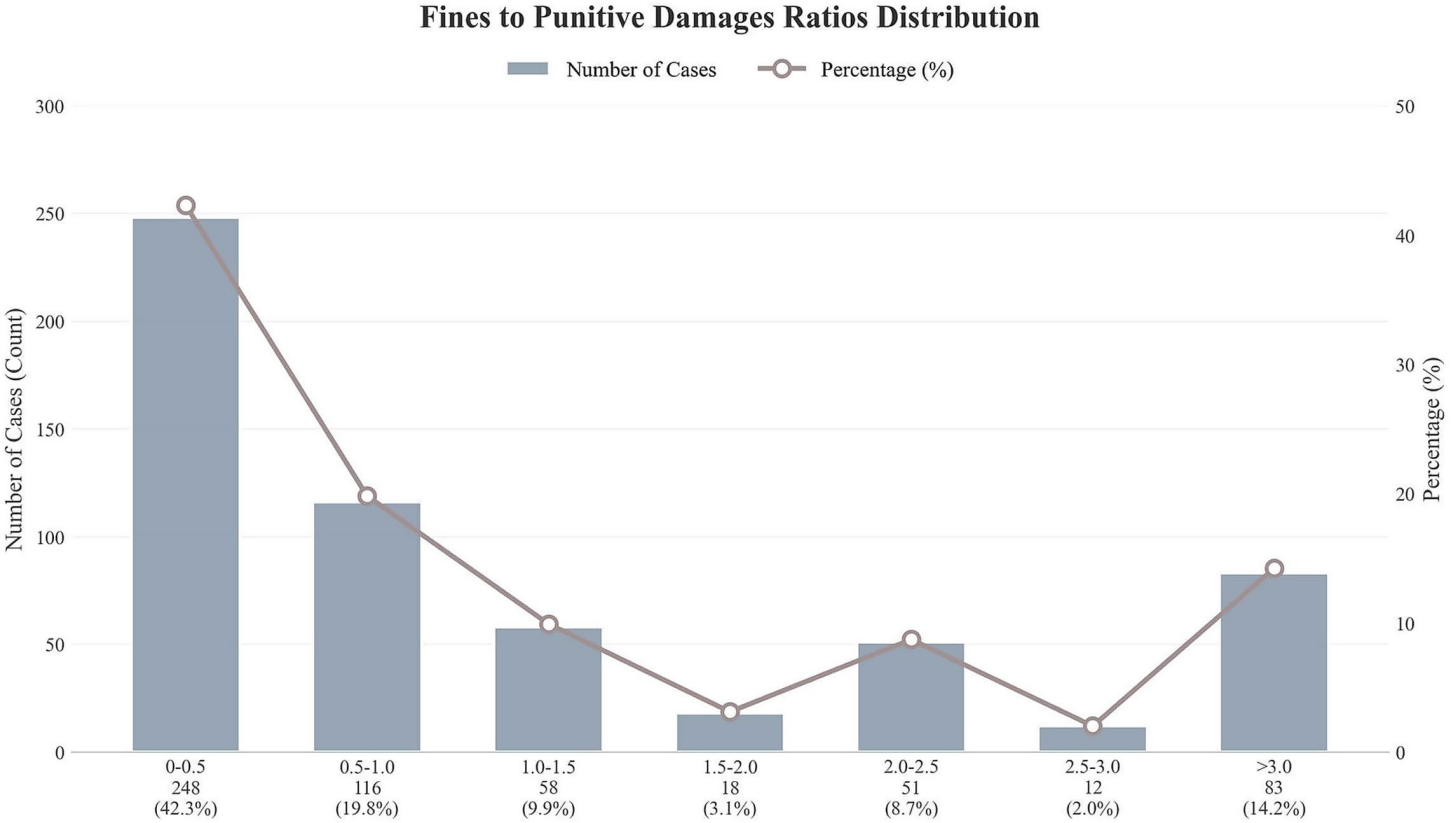
Distribution of punitive damages amounts awarded in 596 cases where the amount was clearly specified. The amounts range from 50 yuan to approximately 42.9 million yuan, showing a highly dispersed distribution. 79.03% of awards were below 100,000 yuan, and 44.30% were below 10,000 yuan.

**Figure 7:**
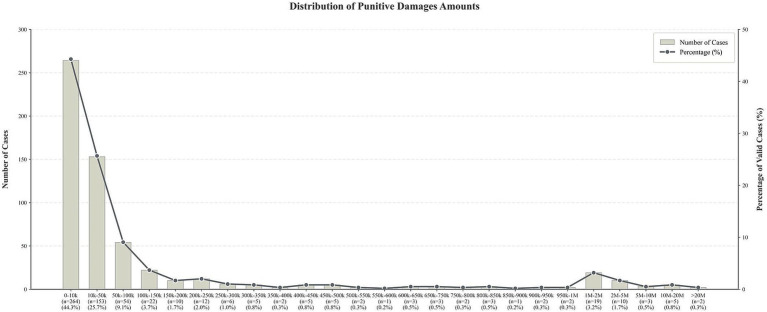
Distribution of the ratio of fines to punitive damages (the “F/P ratio”). The mean ratio (1.766) is significantly higher than the median (0.670), indicating a polarized distribution. In 62.1% of cases, the ratio was below 1, reflecting a pattern of property sanctions dominated by civil liability; in 14.2% of cases, the ratio exceeded 3, reflecting an opposing tendency toward the strengthening of criminal liability.

**Figure 8:**
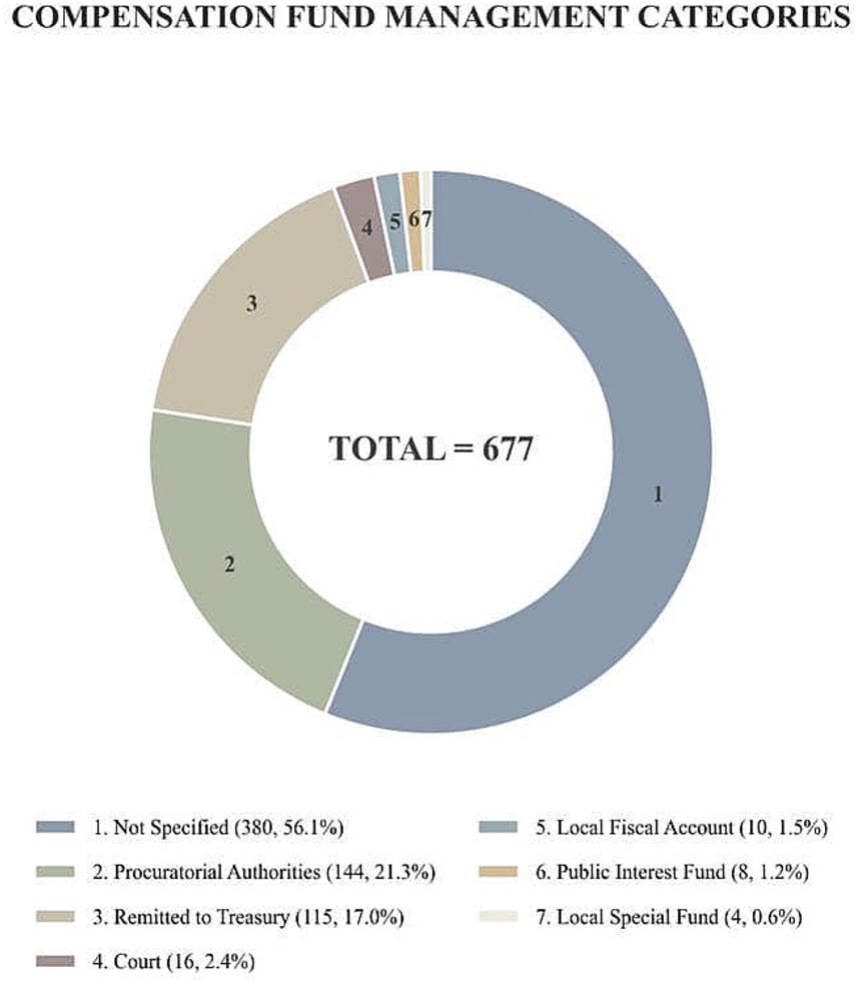
Allocation of punitive damages in cases where the destination was specified. Of these, 40.07% were remitted to local treasuries, and 50.17% were paid to the procuratorial authorities that initiated the public interest litigation. More than half (56.13%) of court judgments did not adequately explain the funds’ destination.

The following figures were generated based on the coded data: geographical distribution of cases (Figure 1), trial level distribution (Figure 2), types of problematic food (Figure 3), pre-litigation announcement website distribution (Figure 4), implementation status of pre-litigation announcements (Figure 5), distribution of punitive damages amounts (Figure 6), distribution of the F/P ratio (Figure 7), and allocation of punitive damages (Figure 8).

## 3 Results

This section may be divided by subheadings. It reports the empirical results of the 677-case sample, interprets their implications, and sets out the key patterns that emerge from the data.

### 3.1 Operational status of criminal incidental civil public interest litigation in food safety

Based on a quantitative analysis of these 677 valid judicial judgments, the operational status of criminal incidental civil public interest litigation in food safety in practice was objectively presented.

#### 3.1.1 Geographical distribution and trial level

From the standpoint of geographical distribution, the cases span 30 provincial-level administrative regions nationwide but shows an uneven spatial concentration (see Figure 1). Hebei Province (47 cases), Shandong Province (44 cases), and Guizhou Province (41 cases) have the highest numbers, together accounting for nearly 20% of the total. High-incidence regions include North China, East China, and Southwest China. In terms of trial level, over 93% of the cases were adjudicated by grassroots courts (see Figure 2), forming a distinctive “decentralized” adjudication structure.

#### 3.1.2 Types of crimes and problematic food

The types of crimes show a clear concentration in terms of the objects and means involved. Between 2021 and the end of 2025, the most common crime was the production and sale of toxic or harmful food (480 cases, accounting for 71%), followed by the production and sale of food that does not meet safety standards (176 cases, accounting for 26%). The remaining cases fell into the following categories: 12 cases of producing and selling subpar or counterfeit goods; 7 cases of selling goods bearing counterfeit registered trademarks; 4 cases of counterfeiting registered trademarks; 3 cases of producing and selling food that does not adhere to hygienic standards; and 3 cases of producing and selling products that do not adhere to safety standards. This distribution pattern is consistent with the retrospective findings of Wang and Li based on data from 1985 to 2019. Their study noted that between 2013 and 2017, the crime of producing and selling toxic or harmful food was already the most common charge, accounting for 60% to 80% of food safety-related criminal cases, while the crime of producing and selling food that fails to meet safety standards was the second most common charge (23). With respect to the specific types of hazardous food products, the infractions showed a strong sectoral concentration. Among the 73 categories of problematic items identified, the top five—namely “fake sexual enhancement products,” “weight-loss products,” “fried dough sticks containing alum,” “problematic meat products,” and seasonings containing poppy shells—accounted for over 75% of all cases (see Figure 3).

#### 3.1.3 Adjudication patterns

An analysis of judicial rulings reveals the following patterns. First-instance courts typically uphold the claims of the procuratorial authorities; in 564 of the sample cases, or approximately 84% of the total, claims were fully upheld. In terms of liability allocation, punitive damages (applied 582 times) and public apologies (applied 497 times) constitute the dominant “compensation + apology” paradigm (with 411 cases involving a combination of both); preventive liability measures such as product recalls, destruction, and consumer warnings were also applied (42 times).

#### 3.1.4 Punitive damages and appellate proceedings

With regard to punitive damages, the procuratorial authorities filed claims against 1,170 defendants (including 31 entities) in 665 cases (with 6 mediation documents incomplete). A total of 1,296 criminal defendants were prosecuted in these cases. Their sentences varied widely. 638 received less than 1 year, 236 received one to 2 years, 53 received two to 3 years, 61 received three to 5 years, 53 received five to 7 years, 10 received seven to 10 years, and 13 received 10 years or more. An additional 209 defendants were sentenced to criminal detention, and 23 were exempt from punishment. Probation was granted to 645 defendants, prohibition orders were issued in 267 cases, and occupation bans were imposed in 41 cases. The court ultimately ordered 1,163 of these defendants to pay punitive damages. In cases where punitive damages were not awarded, three patterns emerged. In three cases, defendants had voluntarily compensated victims before the litigation, so the procuratorial authorities did not file an incidental civil action. In 43 cases, the court rejected monetary claims but ordered non-monetary remedies such as public apologies, product recalls, consumer warnings, or payment of disposal costs. The remaining 23 cases were resolved by mediation or settlement; some agreements did not disclose the terms, some explicitly excluded monetary compensation, and others kept the settlement amount confidential. Notably, the 31 corporate defendants involved were mostly individual businesses, small enterprises, or business units.

Regarding appellate proceedings, only four second-instance cases were identified, yet the reversal rate reached 75%. Two retrial cases, initiated by the procuratorial authorities through appeal, focused primarily on procedural and sentencing issues rather than substantive disputes in the incidental civil public interest litigation.

#### 3.1.5 Summary of operational characteristics

In summary, the practical operation of this system exhibits three key characteristics.

Spatially and procedurally, the overall pattern is one of “broad coverage with a focus on key areas”. While this pattern appears to be driven by geographical factors, it is in fact attributable to a combination of factors, including local judicial initiative, the density of the food industry, and the effectiveness of policy implementation and regulation. At the same time, the highly decentralized adjudication structure reflects the fact that most cases involve relatively minor offenses that do not fall within the jurisdiction of intermediate courts.

Substantively, cases are concentrated in three core areas: first, “functionally deceptive” crimes, represented by fake sexual enhancement products and weight-loss products, which achieve their effects through the illegal addition of banned substances such as sildenafil and sibutramine, posing direct yet covert harm (these two categories together account for over 50% of cases); second, “process abuse” violations, exemplified by fried dough sticks containing alum, reflecting the ongoing problem of excessive additive use in traditional food preparation; and third, “raw material control failures,” represented by problematic meat products and seasonings containing poppy shells, exposing regulatory vulnerabilities in the upstream supply chain and the catering sector. This structure provides clear empirical guidance for implementing tiered and targeted regulatory oversight.

Judicially, current practice exhibits dual characteristics of both a “standardized paradigm” and “preventive expansion.” The allocation of liability centered on “compensation + apology” reflects a remedial philosophy that places equal emphasis on economic sanctions and the restoration of personal dignity. The limited application of preventive liability indicates that the liability framework in judicial practice is evolving from purely ex post punishment toward a comprehensive mechanism that integrates risk prevention, behavioral correction, and public education. The widespread application of punitive damages highlights the role of public interest litigation in providing substantive deterrence. Moreover, it is worth noting that the current focus of adjudication remains on the criminal aspects, while the adjudicative stability of civil public interest litigation is relatively high.

### 3.2 Empirical review of criminal incidental civil public interest litigation in food safety

Based on an analysis of empirical data, the preceding section has provided a broad picture of how the system of criminal incidental civil public interest litigation in food safety operates. To further highlight the practical shortcomings of this system, this section will focus on specific procedures, finding and analyzing typical instances of non-compliance.

#### 3.2.1 Pre-litigation public announcement: formalistic and confined to a single venue

Given the unique nature of criminal incidental civil public interest litigation in food safety cases, which involve the interests of a broad segment of the public, relevant legal provisions emphasize that procuratorial authorities must carry out a pre-litigation public announcement procedure at the initiation stage. The purpose of this preliminary notice is to safeguard the priority right of social organizations and the public to file lawsuits, thereby achieving social coordination in the protection of public interests (24). However, empirical data indicate that, on the one hand, the primary channels through which procuratorial authorities issue pre-litigation public announcements are relatively limited; on the other hand, court judgments often fail to document the status of announcement fulfillment. Analysis of the sample cases shows that no social organizations have applied to join the litigation within the announcement period, suggesting that the pre-litigation announcement procedure may be merely a formality or have been rendered ineffective, and that the mechanism for fulfilling pre-litigation announcements has failed to serve its intended function.

The first issue concerns the internalization and narrowness of public announcement channels. Of the 227 cases where the announcement medium was explicitly specified, 208 (91.7%) published notices on Justice Net and the Procuratorial Daily—both internal platforms within the procuratorial system. The People’s Court Daily and other public media, on the other hand, accounted for 8.3% (see Figure 4). This choice makes management easier and ensures consistency within the system, but the information reaches mostly the legal profession and has a limited impact on the broader public.

A further issue relates to the lack of key details in court judgments. In 32.6% of the sample cases (221 cases), there was no mention of the pre-litigation public announcement procedure. Furthermore, among the 454 cases where the procedure was actually performed, only half (227 cases) specified the announcement medium in the court judgments (see Figure 5). This reveals a high degree of arbitrariness in how procedural matters are documented, as well as a lack of transparency on key aspects of procedural implementation. Such practices make it difficult for external oversight to understand the substance of the proceedings, exposing the procedure to the risk of becoming a closed-loop operation carried out merely for compliance.

A final issue concerns the ineffective engagement of the public through the pre-litigation mechanism. Although no social organizations applied to join the litigation during the announcement period in the sample cases, there were instances where citizens directly filed civil claims for compensation in the course of criminal proceedings. For example, in the case of *Zhang and Ma et al. for the Crime of producing or Selling Toxic or Harmful Food* (Linze County People’s Court, Gansu, 2023), the victim Zhang sought to file an incidental civil action during the criminal trial, claiming tenfold punitive damages. The court ultimately dismissed the claim due to Zhang’s status as a “knowing buyer” (a consumer who purchases with the intent to claim compensation). Yet the very fact that such a claim was brought shows that the public has a demand for compensation in food safety infringement cases, but was unable to participate in the pre-litigation phase due to inadequate public notice. Civil disputes that could have been channeled in advance and resolved through negotiation via the announcement procedure were instead forced into ongoing criminal proceedings. This not only increased the burden of litigation but also undermined procedural efficiency by delaying intervention.

#### 3.2.2 Civil adjudication’s over-reliance on criminal proceedings

During the substantive adjudication stage, the procedural arrangements, trial activities, and standards of proof concerning the civil component all exhibit a deep dependence on the framework of criminal adjudication, forming a highly structured subordinate relationship. First, in terms of procedural sequence, the civil component is in an absolutely lagging and passive position. The order of adjudication is rigidly fixed, with as many as 76.88% of cases (512 cases) strictly adhering to the linear process of “criminal liability first, and then civil liability”. The initiation, progress, and pace of civil public interest litigation are largely determined by the adjudication of the criminal component, placing it in a secondary and subsequent position in the procedural sequence. The constraining effect of trial sequencing on civil remedies is not observed for the first time in this study. A systematic examination of the procedural dependency of civil proceedings attached to criminal cases has already revealed the fundamental pattern whereby civil public interest litigation is constrained by criminal proceedings in terms of both initiation and procedural pace (18). The empirical data presented in this paper further demonstrate that this dependency has become the norm in the vast majority of cases. Second, during courtroom proceedings, the focus of evidentiary hearings and debates is heavily concentrated on criminal conviction and sentencing. In contrast, civil disputes—such as the scope and extent of harm, the form of liability, and the amount of punitive damages—receive markedly less attention and room for debate. Third, regarding standards of proof, there is a tendency to adhere to the stringent standards applied to criminal evidence, making it difficult for evidence required for the civil component—often based on professional assessments and reasonable inferences—to undergo independent scrutiny and be admitted.

#### 3.2.3 Punitive damages and criminal fines: broad judicial discretion and disproportionate ratio

The *Food Safety Law* introduced a punitive damages mechanism known as “refund plus tenfold compensation (a form of punitive damages)”. (25) Empirical findings show that “tenfold” compensation dominates judicial rulings (58.29%); “threefold” compensation serves as an important supplement (18.72%), with rulings frequently citing Article 55 (1) of the Consumer Rights and Interests Protection Law. The statutory minimum compensation standard of “1,000 yuan” also accounts for a significant proportion (13.01%), reflecting the widespread application of sanctions for small-value infringements. In some small-value cases, courts have not mechanically applied the 1,000 yuan floor; instead, they have determined lower amounts based on the overall circumstances, demonstrating flexibility in judicial discretion. However, this formal uniformity has not achieved substantive consistency in adjudicative standards. Genuine differences in judicial discretion are concealed within the adjustment of the base amount and the fluctuation of the multiplier (ranging from 10% to tenfold), which is the main reason for the wide disparity in outcomes among similar cases.

This disparity is reflected in two main aspects.

To begin with, the broad discretion in the imposition of punitive damages has resulted in a highly uneven distribution of awarded amounts. This study collected data on punitive damages in 671 cases, of which 596 (88.82%) had clearly specified amounts (see Figure 6 for details). In 79.03% of these, the amount awarded was below 100,000 yuan (44.30% below 10,000 yuan). When the amount exceeded 500,000 yuan, the number of cases dropped sharply, with only 52 cases (8.72% of the total sample) falling into this range. Punitive damages varied widely, with the highest amount reaching approximately 42.9 million yuan and the lowest only 50 yuan. This range of over 40 million yuan highlights the highly dispersed distribution of punitive damages in food safety cases.

Adding to this is the disproportionate ratio between punitive damages and criminal fines. The ratio of fines to punitive damages—calculated by dividing the total criminal fine by the total punitive damages awarded—reveals the balance between criminal property sanctions and civil penalties for the same unlawful act. In 62.1% of cases, this ratio was less than 1, meaning that the amount of punitive damages was close to or even exceeded the fines, forming a pattern of property sanctions dominated by civil liability (26). In 14.2% of cases, the ratio exceeded 3, with fines far surpassing punitive damages, reflecting an opposing tendency toward the strengthening of criminal liability (see Figure 7). This polarized ratio, combined with the fact that the mean (1.766) was significantly higher than the median (0.670), suggests that the judiciary faces challenges in striking an appropriate balance between criminal and civil property sanctions and has yet to establish a stable, balanced synergy.

## 4 Discussion

Before proceeding to the analysis of institutional dilemmas, it is necessary to situate China’s criminal incidental civil public interest litigation within the global genealogy of collective redress. Worldwide, collective redress mechanisms broadly follow three distinct trajectories.

The first is the U.S. class action model, driven by private lawyers and market logic, which achieves deterrence through punitive damages (27), but at the cost of intensified settlement pressure and significant erosion of damages by attorneys’ fees (28).

The second is the EU representative action model, brought by qualified entities and characterized by ex ante public supervision, which strictly excludes punitive damages (29). The intermediate paths developed by Australia and Canada within the common law tradition can be seen as extensions of this trajectory (30). Specifically, the class action legislation in both countries adopts the “opt-out” default rule, whereby all persons falling within the class definition are automatically included in the proceedings unless they expressly opt out. However, Australian courts have further developed a “closed class” mechanism, requiring class members to register in advance in order to participate in subsequent settlement distributions. This effectively embeds an opt-in element within the opt-out framework, thereby creating a hybrid structure of “opt-out in principle, opt-in as an exception”.

The third trajectory—shared by China and Brazil—is the developing-country model of procuratorial public interest litigation, in which the procuratorate takes the lead and public interest protection is incorporated into state authority (6). The fundamental divergence between the two lies in the fact that the Brazilian procuratorate can bring independent public civil actions and seek preventive injunctions (*Lei n°7.347/85*, Art. 12), whereas China’s civil remedies are procedurally tied to criminal prosecution, creating a path dependence of “criminal priority”.

A comprehensive survey of over twenty jurisdictions worldwide further indicates that the choice between opt-in and opt-out, the legality of litigation funding, and the breadth of the scope of actionable claims are the three core contested issues in the design of collective redress mechanisms (31–34). Opt-in and opt-out respectively, determine class membership and the binding effect of the judgment either by requiring direct and explicit consent from class members or by automatically including them unless they expressly opt out. Opt-out tends to enlarge class size and strengthen deterrence, but offers weaker protection for members’ procedural autonomy; opt-in better safeguards members’ procedural rights, yet may result in under-coverage of collective redress due to “rational apathy.” Different institutional choices reflect a country’s value orientation regarding the structural tension between judicial efficiency and procedural justice.

The evolution of public interest litigation in India precisely illustrates this point. Through “epistolary jurisdiction,” Indian courts intervene in areas of public risk and provide emergency relief: the Supreme Court or High Courts may treat letters, postcards, or news reports from any citizen or social group as writ petitions, thereby initiating public interest litigation proceedings and dramatically lowering the threshold for access to justice. However, precisely for this reason, the number of public interest cases has ballooned, and the scope of accepted cases has expanded excessively, ultimately triggering disputes over the blurred boundaries between the judiciary and the executive—serving as a cautionary tale for other countries in prudently managing the allocation of power.

On this issue, China offers a distinct solution. By granting procuratorial authorities a monopoly on the right to sue as public interest representatives, China has effectively created a state-led alternative to opt-in, while limiting the scope of actionable claims to the meaning of “social public interest.” However, a litigation funding system has yet to be established. China’s system of criminal incidental civil public interest litigation in food safety cases provides a valuable case study for global collective redress mechanisms, and it is practically significant to examine the particularities of China’s issues from a comparative law perspective.

### 4.1 Structural dilemmas of criminal incidental civil public interest litigation in food safety

The various operational difficulties that emerge in the practical operation of a litigation system can often be traced back to unresolved structural contradictions inherent in the system itself (35). In China’s criminal incidental civil public interest litigation system in food safety, these systemic obstacles are mainly reflected in five aspects: the ambiguous definition of the scope of cases at the initiation stage; the conflict between criminal and civil procedures and the conflation of standards of proof at the adjudication stage; the unchecked discretion in the imposition of punitive damages and criminal fines at the judgment stage; and the poor coordination at the enforcement stage.

#### 4.1.1 The dilemma of defining the boundaries at the initiation stage

Whether a public interest litigation is initiated and pursued depends on whether the case involves “social public interest.” Against the backdrop of growing demand for criminal incidental civil public interest litigation in the field of food safety, the vague definition of “social public interest” has, to some extent, weakened the screening function of the procedure. Consequently, the scope of cases has expanded arbitrarily, making this the primary challenge at the initiation stage.

##### 4.1.1.1 The behavior-centric approach to defining “social public interest”

Currently, the absence of a clear legislative definition of “public interest” has not led to prudent case-by-case adjudication in judicial practice^2^. Instead, it has given rise to a simplistic tendency that can be characterized as a “behavior-centric approach.” In the context of criminal incidental civil public interest litigation, this approach means that judicial authorities substitute a finding of illegality for an independent assessment of harm. The academic community holds differing views on the criteria for defining “social public interest.” Some scholars have classified the relevant approaches into five types: “ontological negation,” “holistic interest theory,” “majority interest theory,” “theory of the sum of individual interests,” and “abstract order theory” (36). Among these, the “sum of individual interests” theory maintains that public interest should be reduced to harm to the individual rights of an unspecified majority, while the “abstract order” theory focuses on the maintenance of the legal order itself. The “behavior-centric” tendency identified in this study essentially equates the procedural requirements for determining the maintenance of legal order with the evidentiary requirements for proving harm to the public interest, using the former’s conclusion of unlawfulness to obscure the independent duty to review the latter. Once an act is confirmed as a criminal offense or an administrative violation, it is presumed that the civil element of “harm to social public interest” is automatically established, thereby bypassing any independent review of whether harm actually exists, its specific nature, and its causal link (37). This tendency is specifically manifested in the following two types.

The first type equates abstract regulatory risks with concrete public interest harm. Some judicial opinions hold that any conduct violating mandatory administrative regulations in the food safety field (such as quarantine procedures or import permits) constitutes an infringement upon social public interest, regardless of whether such conduct actually endangers the life, health, or property rights of an unspecified number of consumers^3^. For example, in the case of *Fu Zejun et al. Selling Food Not Meeting Safety Standards*^4^, the frozen products in question passed inspection; yet, solely because they lacked legal import documentation, the court ruled that public interest had been harmed on the grounds of “disrupting the national epidemic prevention and control order.” In other words, such rulings use administrative violations as a substitute for the elements of civil tort, thereby confusing the legal interests protected by different branches of law.

The second type is even more typical: the criminal conviction supersedes the independent proof of civil harm. After a criminal judgment is rendered, the civil public interest litigation part is often disposed of with a single formulaic sentence—“the act constituted a crime, therefore public interest was harmed”—without any further evidentiary support or discussion of the specific facts or extent of the harm. A telling illustration is found in the cases of *Pang Wei Producing and Selling Toxic or Harmful Food*^5^ and *Ma Yisifu Selling Toxic or Harmful Food*^6^. There, the criminal convictions were directly substituted for the required proof of actual harm or specific danger to an unspecified number of consumers. Based solely on the finding of an abstract, dangerous crime at the criminal level, the courts inferred that the type of danger had been established, thereby justifying punitive damages in civil liability. This severely curtailed the space for civil adjudication.

##### 4.1.1.2 The insufficient utilization of the screening function at the initiation stage

From the perspective of litigation principles, the scope of cases admissible for procuratorial public interest litigation should be subject to two limitations. First, there must be a substantive necessity for the procuratorial authorities to initiate such litigation. Second, as a last-resort judicial remedy, there must be a pressing practical need arising from the lack of other available avenues for redress (38). The former is conditioned on the court’s substantive judicial review of whether “social public interest has been harmed,” while the latter depends on the effective operation of the pre-litigation public announcement procedure as a prerequisite for the right of action. However, empirical findings reveal two problems.

First, the courts’ simplistic behavior-centric approach to determining harm to social public interest has considerably weakened the screening function of the system. This has allowed a large number of cases—those involving only formal illegality (such as labeling defects or incomplete formalities) or extremely vague or minimal harm to social public interest—to enter the scope of criminal incidental civil public interest litigation. Consequently, the effective check that the People’s Courts could exercise over procuratorial authorities’ acceptance of public interest litigation has been undermined, leading to a genuine risk of lower thresholds for initiating litigation and arbitrary expansion of the scope of cases.

Second, given that procuratorial public interest litigation is characterized by high litigation costs and significant consumption of judicial resources, its initiation by the procuratorial authorities should be premised on the practical necessity of having exhausted other judicial remedies—that is, it should serve as the last line of defense for the protection of litigation interests. Yet, the hollowing out of the pre-litigation public announcement procedure, coupled with the lack of effective external oversight, has left the procuratorial authorities as the absolute discretionary authority in initiating public interest litigation. This may result in the failure to protect the litigation interests of private rights holders, causing other remedies to wither or even be displaced. Ultimately, this leads to the undesirable situation where public interest litigation is unduly expanded, judicial resources are not effectively utilized, and the public interest is not fully protected.

#### 4.1.2 The dilemma of procedural arrangements in case adjudication

In the Chinese context, criminal incidental civil litigation has evolved into a situation in which three models coexist: “criminal proceedings first, then civil proceedings,” “separation of criminal and civil proceedings,” and “civil proceedings first, then criminal proceedings” (39). The “criminal first” model prioritizes the determination of criminal liability and the uniformity of adjudicative outcomes; the “separation” model insists that the two procedures run independently; and the “civil first” model prioritizes the timeliness of civil remedies. The 1979 Criminal Procedure Law initially established the “criminal first” trial sequence at the inception of the system. Under the long-standing judicial emphasis on punitive justice, the dominant position of criminal procedure was gradually strengthened, and “criminal first” became the mainstream practice.

Article 20 (2) of *the Interpretation of the Supreme People’s Court and the Supreme People’s Procuratorate on Several Issues Concerning the Application of Law in Public Interest Litigation Cases* (2018) explicitly provides: “Cases of criminal incidental civil public interest litigation initiated by the People’s Procuratorate shall be under the jurisdiction of the People’s Court that hears the criminal case.” Although this provision resolves the jurisdictional disputes in food safety public interest litigation, it fails to eliminate the subordination of civil public interest litigation once it enters the adjudication stage. On the contrary, this subordination is further entrenched by the institutional framework of “criminal incidental civil litigation.” The timing of initiating civil remedies, the scope of factual determinations, and the pace of adjudication are all dominated by criminal logic, reducing the public interest litigant from a procedural leader to a passive waiter (30). This high degree of procedural entanglement triggers a series of negative consequences.

##### 4.1.2.1 Civil adjudication demonstrates high subordination

Generally speaking, the fundamental adjudicative logic of criminal incidental civil litigation is “criminal liability first, and then civil liability.” This approach is primarily based on considerations such as the priority of criminal liability, procedural efficiency and convenience, and smooth procedural coordination. In certain exceptional circumstances, alternative approaches like “civil liability first, then criminal liability” or “parallel criminal and civil proceedings” may be considered. However, the sample cases show that courts rarely deviate from this rigid order, even when the civil dispute is extremely straightforward and fully capable of being adjudicated independently. Consider, for instance, the case of *Li Yuanwang Selling Food That Does Not Meet Safety Standards*^7^, where the civil claim was merely the sales amount itself, 2,700 yuan. The facts were clear, and the defendant expressed a clear willingness to mediate. The case could have been resolved through mediation before or during the criminal trial. Yet it was still forced to the end of the criminal process as an “incidental” matter, thereby suppressing civil relief.

##### 4.1.2.2 Resulting in failed incentives and delayed remediation

Procedural bundling gives rise to numerous issues, including insufficient incentives and delayed remediation. Incentives are undermined in the first place. The rigid application of the “criminal liability first, and then civil liability” principle makes it difficult to obtain timely confirmation of civil compensation and convert it into sentencing leniency, potentially causing defendants to miss the optimal opportunity to make an informed decision to plead guilty and accept punishment, as well as to secure procedural benefits under lenient treatment. Remediation, in turn, is delayed. One of the unique institutional values of public interest litigation lies in its ability to effectively intervene in the ongoing risks posed by illegal activities such as food safety violations. Rigid adherence to the “criminal liability first, and then civil liability” sequence can easily lead to excessive delays in litigation. The window of opportunity to stop harm and eliminate risks is constrained by the criminal trial cycle and is unfortunately lost to it, causing the scope and severity of the public interest damage to expand accordingly. Furthermore, the enforcement of the final judgment may be undermined by circumstances such as the defendant’s transfer of assets or deterioration of their ability to pay (40).

#### 4.1.3 The dilemma of conflated standards of proof

The negative impact of the tight coupling of criminal and civil procedures during court proceedings is not only evident in the hollowing out of judicial reasoning in the civil portion—where the focus of adjudication and litigation resources are excessively skewed toward the criminal part, resulting in the widespread compression of civil dispute adjudication—but also gives rise to a further difficulty: the strong subordination of civil public interest litigation leads to a dilemma in judicial proof. From the standpoint of evidentiary norms, the joinder of civil and criminal proceedings inherently creates conflicts in the coordination of judicial proof. Criminal proceedings center on conviction and sentencing, adhering to strict evidentiary rules and the standard of “proof beyond a reasonable doubt.” Civil public interest litigation, by contrast, aims at remedying harm and restoring order, following the standard of “preponderance of the evidence” and allowing reasonable inferences based on the weight of the evidence. In the context of criminal incidental civil public interest litigation in food safety, this manifests as a “compatibility” or “conflation” of the two standards of proof—or more precisely, as criminal evidentiary rules overriding the civil part (41).

##### 4.1.3.1 Using the criminal amount as the basis for civil compensation

In criminal cases, the amount involved must be supported by precise evidence and thorough corroboration. In civil public interest cases, by contrast, a comprehensive assessment of multiple factors is required—such as the duration of sales, product distribution, and the scope of potential risks. In practice, most calculations of punitive damages lack such a comprehensive evaluation and simply use the sales amount established in the criminal proceedings as the base. For example, in the case of *Ren Hongqin, Niu Yuquan, et al., for the Crime of Producing or Selling Food That Does Not Meet Safety Standards*^8^, the court strictly distinguished between sales proceeds and private loans. As criminal evidentiary standards became increasingly stringent, the basis for civil damages became increasingly fixed on the amount of goods sold, thereby narrowing the overall harm of the conduct to the criminal amount. The crux of the problem lies in quantifying harm—such as health risks—in food safety public interest litigation. This quantification depends on expert reports that estimate overall risk. However, such reports, based on statistical and probabilistic principles, have a “speculative” nature. During criminal evidence review, they often face challenges to admissibility due to their “uncertainty.” Moreover, at the institutional level, no independent review channel has been established for such expert reports, so they are frequently excluded on grounds of speculation (42). For instance, in a series of cases involving the illegal addition of sildenafil^9^, courts could determine the criminal amount based on sales ledgers; yet, there was no procedure in place to introduce or cross-examine expert reports assessing systemic health risks.

##### 4.1.3.2 Substituting criminal knowledge for civil fault determination

At the subjective level, the determination of “knowledge” as an element of a criminal offense and the evaluation of “fault” in civil unlawful acts directly affect the outcome of judicial decisions. In practice, however, courts have failed to distinguish between the two during trial proceedings; the finding of criminal knowledge is often directly applied as the basis for assessing civil fault. This approach of conflating “knowledge” with “fault” overlooks the nuanced hierarchy of fault forms within the civil tort liability system and fails to recognize that different forms of fault—such as intent and gross negligence—can lead to different adjudicative outcomes. Take the case of *Xiong Moumou*^10^ as an example. After being warned of the risks, the defendant still failed to perform his duty of inspection and was found to have acted with indirect intent. During the civil proceedings, the court neither restated the facts of fault nor distinguished between the different forms of fault. Instead, it simply relied on the criminal finding that “Xiong Moumou knowingly sold food that did not meet food safety standards” as the basis for ordering him to pay tenfold punitive damages. This reflects that in criminal incidental civil public interest litigation in food safety, courts lack independent review and careful differentiation of civil fault forms, thereby reducing the room for discretion in the subjective assessment of civil unlawful acts.

#### 4.1.4 The dilemma in the application of punitive damages

Traditional punitive damages are structured around the individual consumer as the right-holder, take actual harm as the basis for calculation, and pursue the dual objectives of private interest compensation and behavioral deterrence. Their doctrinal foundation rests on a bilateral liability structure of “the wrongdoer compensating the victim,” essentially providing additional solace for the specific harm suffered by the particular victim, and fundamentally adhering to a framework premised on the convergence of the right-holder, the beneficiary, and the party bearing the harm within the same subject (43). By contrast, public interest punitive damages detach the right to claim from the victim. They no longer respond to private moral condemnation but address the systemic erosion of consumer order and public safety caused by mass torts. Their core function thus shifts from individual solace to the pricing and punishment of systemic risk. Public interest punitive damages have thus evolved into an independent institutional framework: the right-holder shifts from individual consumers to the procuratorial authority; the beneficiary expands from specific victims to an unspecified group of consumers; and the institutional objective shifts from compensating private losses to restoring the disrupted consumer order and food safety environment (44).

Although public interest punitive damages exhibit distinctive characteristics in terms of the basis of the claim, the nature of liability, and the allocation of damages, the sample cases reveal negative patterns such as wide disparities in the amounts awarded, an imbalance between criminal and civil liability, and inconsistent outcomes in similar cases. Based on an analysis of 1,271 judgments, Zhu and Zhao (45) attributed these phenomena to deficiencies such as a lack of discretionary mechanisms, the absence of enforcement measures, and gaps in the management system for the receipt of funds. The empirical analysis in this paper further demonstrates that these shortcomings do not exist in isolation but are closely linked to the procedural structure of criminal incidental civil public interest litigation, manifesting primarily as systemic challenges such as doubts regarding the legitimacy of the right-holder, ambiguous standards of discretion, and the absence of rules for allocating liability.

##### 4.1.4.1 Legitimacy dispute over the claiming party

Whether procuratorial authorities have the authority to claim punitive damages—which are inherently the right of consumers—constitutes a fundamental prerequisite and obstacle to the functioning of the system. Both Article 148 of the *Food Safety Law* and Article 55 of the *Consumer Rights and Interests Protection Law* grant the right to claim punitive damages to “consumers,” with the legislative intent being to provide remedies for private interests. However, Article 58 of the *Civil Procedure Law* and the *Rules for the Handling of Public Interest Litigation Cases by the People’s Procuratorates*, which serve as the primary procedural bases for public interest litigation, do not explicitly grant procuratorial authorities this substantive civil right. Consequently, procuratorial authorities lack clear authorization under higher-level laws when directly claiming punitive damages in public interest litigation. In contrast, judicial practice has established a “custom without legal basis”: procuratorial authorities act as the sole claimants and receive a high rate of court support (88.7%, of which 84.2% were fully upheld), effectively substituting for the statutorily designated claimants (6).

The root of the dispute over the right-holder lies in the institutional differences between public interest punitive damages and private interest punitive damages. The core functions of punitive damages are generally summarized as punishment, deterrence, and retribution (46). In the private interest punitive damages system, the right-holder is always the individual consumer who has suffered actual harm; the functional relationship is relatively stable, and the issue of who is the right-holder has never risen to become a central controversy. However, in the context of public interest punitive damages, the claimant shifts from the consumer to the procuratorial authority, resulting in a separation between the right-holder and the beneficiary. This separation renders the existing framework for private compensation incapable of accommodating such a structure where the right-holder and the beneficiary are distinct.

In response to this controversy, two interpretive approaches have emerged at the theoretical level. The “derivative right of action in public interest litigation” doctrine holds that when unlawful acts infringe upon the rights and interests of an unspecified number of consumers, such acts independently harm the social public interest (47). Procuratorial authorities, acting on their statutory duties, claim punitive damages as an independent right to public interest relief. Under this view, the function of punitive damages shifts from compensating specific consumers to deterring unlawful conduct and restoring the damaged public interest (44). Most judicial decisions adopt this stance. The “statutory private right of action” theory, by contrast, challenges this approach, emphasizing that punitive damages are private rights granted by law to “consumers” as specific civil subjects, adhering to the principle of statutory rights of action (19, 48). Procuratorial authorities are not consumers, and directly claiming such damages lacks a substantive legal basis, potentially blurring the procedural boundary between public and private law (49). The conflict between these two interpretations raises a fundamental question: when punitive damages are introduced from the realm of private law into public interest litigation, what transformation occurs in their legal nature? Do they become a public interest instrument, or remain a private right application?

##### 4.1.4.2 Unchecked discretion in determining the amount of punitive damages

The issue of “who is entitled to claim punitive damages” lies at the heart of standing disputes. However, once it is accepted that procuratorial authorities may claim such damages, the immediate challenges become “how much to award” and “how to allocate liability.” The data presented above show a high dispersion of punitive damages amounts and a polarization of the ratio of fines to punitive damages, indicating a lack of uniform rules in judicial practice for determining amounts and allocating liability. Compared to the standing issue, the unchecked discretion in determining the amount of punitive damages has become a more intractable institutional dilemma.

First, the lack of adversarial structure in litigation fundamentally undermines the procedural constraints on judicial discretion. In practice, as many as 62.6% of defendants did not raise substantive objections to claims for punitive damages, focusing their defense instead on securing sentencing leniency. Furthermore, although the vast majority of cases (88.8%) involved retained counsel, their defense strategies were significantly skewed toward sentencing (accounting for 54.5%), and it was rare for them to raise professional challenges to punitive damages claims in terms of standing, factual findings, or the application of law. This strategy of “trading civil compromise for criminal leniency” means that key issues—such as the calculation base and multiplier of damages—go uncontested and undebated in court. Judicial review, lacking adversarial checks, is highly prone to devolving into a unilateral confirmation of the prosecution’s claims.

Second, insufficient judicial review by the People’s Courts further contributes to the arbitrariness of the amount awarded. In the few cases where courts reduced the amount of punitive damages, their reasoning was often highly generalized, employing non-standardized phrases such as “the defendant’s inability to pay,” “based on the overall circumstances,” or “in light of the actual situation.” Such reasoning fails to explain clearly why the statutory tenfold damages were reduced to one or two times, and it is even less capable of explaining why similar unlawful acts in similar cases led to vastly different multipliers. Moreover, as to the “sales amount” used as the calculation base, courts differ on whether to include ancillary costs such as transportation and storage, and whether to accept the prosecution’s alleged amount or the defendant’s self-reported amount. For example, regarding the inclusion of ancillary costs, in the *Wu* case, the court reduced the sales amount alleged by the procuratorial authorities from over 220,000 yuan to just over 130,000 yuan, excluding courier fees. Regarding the standard for admitting evidence, in the *Xu* case, the court accepted the defendant’s self-reported lower amount as the calculation base on the ground that “downstream buyers had not been identified”.^11^ Furthermore, the lack of effective appellate supervision exacerbates these issues. The few appellate reversals are primarily driven by criminal policy considerations (e.g., guilty pleas, acceptance of punishment, or restitution) or corrections of individual factual findings. For example, in the case of *Cheng Yumeng*, the appellate court modified the sentence to probation based on the defendant’s guilty plea, acceptance of punishment, and active performance of compensation—an adjustment driven by criminal policy considerations. In the case of *Wang Moujia*, although the appellate court clarified the rules for determining the calculation base of punitive damages, this was merely a correction of an error in the first instance’s factual determination and did not address deeper issues of discretion, such as the determination of the multiplier. Neither case provided a systematic or general interpretation of the discretionary rules for punitive damages^12^. As a result, the disorderly use of discretion at the trial level goes unchecked, ultimately leading to the adverse situation of “different judgments for similar cases” and a lack of uniformity in judicial decisions.

Third, the ineffective application of mediation and settlement procedures correlates with the misuse of discretionary power. In the sample, only 13.00% of cases were concluded by mediation or settlement. The blockage of negotiated channels means that many cases that could have achieved public interest repair through pre-litigation or pre-trial agreements instead flood into the adjudication process. Procuratorial authorities, constrained by their role as public interest representatives, take a cautious stance toward mediation and withdrawal. One problem is that the space for mediation is limited, often confined to the monetary claims already stated in the indictment, featuring formalized negotiation^13^. Another problem is that withdrawal is rarely available and subject to strict conditions; in the entire sample, only three cases ended with the procuratorial authorities withdrawing, and all were contingent upon the defendants fully performing the monetary obligations already demanded^14^. Even in the few cases where mediation was initiated, there was no stable or transparent mechanism linking the negotiation and performance of agreements to criminal sentencing considerations. Defendants could not secure clear consideration of their sentences through proactive restorative actions. When the path of negotiation narrows, adjudication becomes the only option, and the discretion over punitive damages is placed under pressure without any diversion or buffer.

##### 4.1.4.3 Confusion in the allocation of liability under special circumstances

In cases involving multiple tortfeasors or multi-level distribution chains, the allocation of liability for punitive damages faces complex conflicts among civil tort principles, criminal complicity theories, and the purposes of public interest litigation, leading to chaotic outcomes in practice. Three particular issues stand out.

One concerns the overly simplistic division of joint and several liability. In cases of joint criminal activity, courts generally hold co-defendants jointly and severally liable but fail to make a precise allocation of their respective shares of liability. A common practice is to directly use the criminal classification of principal and accessory to allocate—or even exempt—civil liability; for instance, requiring only the principal to bear the full amount of punitive damages^15^. This confuses the key focus of criminal liability assessment (the role of the act in the crime) with the basis for attributing civil liability (fault and causation).

Another issue concerns the ambiguous scope of liability in multi-tiered distribution chains. In a sales network comprising multiple stages—such as production, wholesale, and retail—there is no uniform standard as to whether the compensation base should be the end-user sales price or the price at each level of resale (50). If each level is ordered to pay punitive damages based on its own sales amount, the same batch of products may be subject to duplicate assessments of harm, resulting in an unreasonably high total compensation that deviates from the principle of proportionality between offense and punishment. Some judgments attempt to limit the liability of upstream sellers to the “downstream sales amount” or “purchase price,”^16^ but the choice of benchmark and its legal basis are often inadequately explained, leaving significant room for arbitrary discretion.

In addition, consumer fault is emerging as a new factor affecting the application of punitive damages (47). In certain cases, courts or procuratorial authorities may occasionally exclude or reduce punitive damages on the grounds that the consumer engaged in “knowing buying” (professional claims) or that the consumer’s own conduct was illegal (e.g., consuming protected animals)^17^. In handling such cases, judicial authorities must navigate a difficult balancing act between, on the one hand, punishing unlawful operators and protecting the public interest in food safety, and, on the other, preventing abuse of the system and safeguarding other legally protected interests (such as market integrity and ecological protection). However, no consistent discretionary benchmark has yet emerged for such balancing, further exacerbating the unpredictability of adjudicative outcomes.

#### 4.1.5 The dilemma of coordination in judgment enforcement

An examination of whether the criminal incidental civil public interest litigation system operates smoothly does not end with the final issuance of a judicial ruling. At the enforcement stage, the effective realization of the system’s objectives is hindered by two obstacles: one is the conflict with the enforcement of criminal fines, and the other is the disorderly subsequent management of the punitive damages. The former concerns “whether enforcement can be fully carried out,” while the latter determines “whether the system’s objectives can be achieved after enforcement”.

##### 4.1.5.1 Unclear order of payment between criminal fines and punitive damage

In food safety-related criminal incidental civil public interest litigation, it is common for perpetrators to be sentenced to both criminal fines and punitive damages. Although both involve monetary sanctions, they fall under criminal penalties and civil liability, respectively, and are fundamentally different in nature. Whether the two can be offset against each other and how the order of payment should be determined remains to be clarified. On the issue of offset, judicial positions are sharply divided. While some court rulings explicitly acknowledge that “punitive damages may be offset against fines”, ^18^others reject offset on the grounds that it “lacks a legal basis”^19^ due to the different nature of liability. This phenomenon of inconsistent rulings in similar cases undermines the uniformity of judicial standards. Regarding the order of payment, although Article 187 of the Civil Code establishes that civil liability takes precedence, only a very small number of cases in the sample explicitly address the enforcement sequence. Furthermore, since criminal fines and public interest compensation funds are subject to different enforcement procedures and lack mandatory institutional coordination, enforcement authorities in practice mostly follow general debt repayment rules such as “seizure priority” or “pro rata distribution”. When the debtor’s assets are limited, the combined effect of the absence of offset rules and disorderly payment creates an enforcement deadlock, thereby causing both the public interest repair objective of punitive damages and the deterrent and preventive functions of criminal fines to fail.

##### 4.1.5.2 Inadequate oversight of the allocation of punitive damages

Regarding the follow-up oversight mechanisms for punitive damages—specifically, who manages them and where they are allocated—there is a lack of clear regulatory guidance. On the one hand, the allocation of these funds lacks external oversight. When the destination of the funds was clear, punitive damages were primarily remitted to local treasuries (40.07%) or paid to the procuratorial authorities that initiated the public interest litigation (50.17%). However, more than half (56.13%) of court judgments did not adequately explain the funds’ destination (see Figure 8). In essence, punitive damages in public interest litigation are not part of the state budget, and remitting them to the treasury does not comply with the requirements of the State Treasury Regulations (14). Punitive damages arising from criminal incidental civil public interest litigation in food safety serve the public interest of consumer protection; transferring them to the treasury does not help address issues such as the disruption of consumer order caused by operators’ illegal acts. As for the alternative route, although the funds are nominally held in trust by the procuratorial authorities, the absence of statutory mechanisms for follow-up oversight and dedicated use means that the funds either remain inactive in agency accounts or are ultimately transferred to the treasury. In either case, there is a “disconnect” between the punitive damages and the damaged public interest, making it impossible to allocate them directly and leaving them beyond the scope of substantive protection for social public interest in food safety. On the other hand, professional management mechanisms are lacking. Most regions have not established dedicated consumer public interest fund accounts, and cases with clearly designated public interest purposes account for only about 3.25% of the total, leading to disorderly fund administration. Given that public interest litigation often involves costs such as expert appraisals, public announcements, and investigations, if punitive damages cannot form a virtuous cycle that recoups litigation costs and supports sustained public interest protection, the sustainability of the public interest litigation system itself will be seriously undermined.

### 4.2 Pathways to optimization for criminal incidental civil public interest litigation in food safety

Overall, structural challenges arise at every stage—from initiation to enforcement—under various circumstances. The refinement of criminal incidental civil public interest litigation in food safety urgently requires a systemic overhaul at the level of coordination between procedural frameworks and substantive rules. Specifically, on the procedural side, the focus should be on breaking the entrenched pattern of “criminal liability first, and then civil liability,” achieving full-process optimization—from case initiation, to adjudication, to supporting mechanisms. On the substantive side, it is necessary to clarify the boundaries between criminal and civil standards of proof and to unify the discretionary standards for punitive damages.

#### 4.2.1 Procedural coordination: from “criminal proceedings first” to a flexible choice of trial models

##### 4.2.1.1 Analysis of the three models: “criminal proceedings first,” “equal emphasis on criminal and civil proceedings,” and “civil proceedings first”

The “criminal proceedings first” model pursues the priority of criminal adjudication, and its legitimacy rests on three core legal grounds. First, the primacy of state penal power: criminal prosecution serves the public interest and, in the hierarchy of values, takes precedence over civil litigation aimed at private rights remedies. Second, the hierarchical relationship of evidentiary standards: the criminal standard of proof—“beyond a reasonable doubt”—yields more conclusive factual findings, and adopting the “criminal first” approach helps prevent contradictory judgments. Third, the pursuit of procedural economy: having the same adjudicatory body handle civil compensation as an incidental matter avoids the resource waste of dual proceedings (51). These legal grounds are self-consistent in purely private-interest incidental litigation, but a logical rupture occurs when public interest litigation enters the incidental framework. The civil public interest claim itself carries public interests such as the life, health, and safety of an unspecified number of people, and its normative status is not necessarily inferior to that of criminal sanctions (52). In the system of criminal incidental civil public interest litigation in food safety, rigid adherence to the “criminal proceedings first” concept may result in the failure to protect the public interest.

The “equal emphasis on criminal and civil proceedings” approach advocates that criminal prosecution and civil remedies should proceed in parallel, with the two procedures maintaining relative independence from each other and not substituting for one another in terms of standards of proof, evidentiary rules, or procedural progression (53). This approach attempts to make corrections within the existing framework, but it does not fundamentally change the subordinate relationship of civil remedies to criminal proceedings. In practice, it remains difficult to escape the inertia of criminal priority, and the pace of public interest repair can easily be constrained by the progress of culpability determination. In criminal incidental civil public interest litigation in food safety, the “equal emphasis” approach should not serve as the dominant model; it is better suited as a fallback option when the “civil proceedings first” model is difficult to implement.

The “civil proceedings first, then criminal proceedings” model fundamentally readjusts the sequence of proceedings by prioritizing the determination of civil liability and then treating compliance as a factor in sentencing. This transforms civil remedies from an adjunct to criminal prosecution into a prerequisite for sentencing. Its legitimacy is rooted in the principle of the primacy of civil liability. Since the defendant’s criminal and civil liabilities arise from the same act and cannot be entirely separated, resolving civil liability first and using proactive compensation as a sentencing incentive ensures that both types of liability are handled in a balanced manner within a unified framework, thereby avoiding the perceived unfairness of “being punished first and then having to pay compensation again” (54). At the procedural level, since the sentencing standardization reform in 2010, the relative separation of conviction and sentencing procedures has been gradually established. The 2020 “Opinions on Several Issues Concerning the Standardization of Sentencing Procedures” further requires courts to ensure the relative independence of sentencing procedures during trials, thereby providing institutional space for “introducing civil public interest litigation after conviction but before sentencing” (55). Looking abroad, Italy’s 2022 Cartabia reform established the principle of separation between civil remedies and criminal proceedings. The Italian Supreme Court subsequently confirmed in Decisions No. 12507 and No. 15797 of 2025 that civil parties are not absolutely bound by criminal rulings of inadmissibility or acquittals and may independently seek civil remedies. Japan’s system of orders for damages, meanwhile, permits criminal judges to proceed with the civil phase following the conclusion of the criminal trial, treating the fulfillment of compensation as a key sentencing factor. These two approaches collectively reveal a new perspective on the sequencing of criminal and civil proceedings: civil remedies are not necessarily constrained by criminal liability, and it is necessary to decouple the two based on specific circumstances.

##### 4.2.1.2 Flexible selection of trial models based on different circumstances

In cases where the defendant pleads guilty, priority should be given to the “civil proceedings first, then criminal proceedings” sequence. This is because, in the field of food safety, the immediacy and concealment of food consumption mean that once harm spreads, it is difficult to reverse. Defendants have the best knowledge of sales channels and product flows related to the illegal act. Voluntary performance of compensation by the defendant is more effective than compulsory enforcement after judgment, as it can promptly eliminate harmful consequences and restore the disrupted consumer order.

When urgent intervention is required, flexible measures under the “equal emphasis on criminal and civil proceedings” model—such as behavioral preservation measures—may be used. In cases requiring immediate cessation of harm or elimination of danger, courts may explore issuing rulings on behavioral preservation or advance execution upon application before criminal liability is finally determined. For civil compensation claims where the facts are clear, courts should encourage mediation or partial performance at appropriate stages during criminal proceedings, and take the defendant’s proactive efforts to restore the public interest as a significant mitigating factor in sentencing, thereby incentivizing timely assumption of responsibility (20).

In cases where the defendant does not plead guilty or where the case may result in an acquittal, the “criminal proceedings first, then civil proceedings” model still applies. The defendant’s criminal liability is placed in the primary order of evaluation, and a full retrospective assessment of criminal culpability is carried out, so as to avoid the risk of wrongful conviction that may arise from errors in factual findings.

In summary, in the system of criminal incidental civil public interest litigation in food safety, the trial model should be determined according to the specific applicable scenario, breaking the rigid operation of “criminal proceedings first, then civil proceedings” and constructing a flexible, remediation-oriented trial mechanism in which “civil proceedings first, then criminal proceedings” is the primary model, supplemented by the traditional “criminal first” and “equal emphasis” models.

##### 4.2.1.3 Procedural adjustments under the concept of “equal emphasis on criminal and civil proceedings”

At the initiation stage, clear standards for assessing harm to social public interest should be established to correct, at the source, the mindset that “an illegal act automatically implies harm to social public interest”. Procuratorial authorities must bear the specific preliminary burden of proof regarding harm to social public interest, submitting evidence to demonstrate that the illegal act has caused actual harm or a specific danger; they may not rely solely on the criminal unlawfulness of the act as a basis (26). The U.S. Supreme Court made clear in *Dukes*^20^ that class certification requires a preponderance of the evidence showing that all class members have suffered a common injury that is capable of being aggregated on a class-wide basis. The determination of illegality and the proof of harm are established as two independent judicial review obligations; the former does not automatically substitute for the latter—a principle from which we can draw. At the same time, the scope of public interest litigation must be determined by returning to the origins of the system. When exploring new areas, the principle of being proactive and prudent should be upheld, preventing both undue expansion of the scope and excessive conservatism (56). Following this approach, we may attempt to implement typified evidentiary guidance to achieve differentiated review: for cases involving substantive risks, such as illegal additives, it is necessary to prove that specific hazardous substances have entered circulation; for cases involving procedural violations, such as labeling defects, it is necessary to preliminarily establish a scientific connection between the procedural defect and specific health risks. Furthermore, to ensure uniformity in judicial standards, it is recommended that the highest judicial authorities issue guiding precedents and explore the establishment of a regular mechanism for consulting judicial technical experts to assist courts in making scientifically sound determinations of specialized facts.

At the coordination stage, efforts should be made to strengthen the effectiveness of pre-litigation public announcements and mediation mechanisms, enabling forces outside the procedural framework to genuinely participate. The preliminary investigation procedure conducted by Brazilian prosecutorial authorities before initiating public civil actions offers a useful reference for injecting dual functions of screening and incentivization into the pre-litigation public announcement. During the announcement period, not only should other eligible entities be allowed to assert their right of action, but the prosecutorial authorities should also be permitted to issue corrective recommendations to the prospective defendants. Where the defendant proactively repairs the harm and eliminates the danger before litigation, the procedure may be terminated, or such conduct may be taken as an important factor in deciding not to prosecute. On this basis, the announcements should break free from the constraints of internal platforms and be simultaneously published on public channels such as provincial-level legal newspapers or government portals to ensure information accessibility. The content of announcements must uniformly include core elements such as the basic facts of the case (with anonymization), preliminary findings of harm to the public interest, and the means of exercising rights, thereby providing clear guidance for social participation. The fulfillment of the announcement procedure should be made a mandatory part of case files and subject to judicial scrutiny to prevent it from becoming a mere formality. With respect to mediation, it should be clarified that procuratorial authorities, after the criminal facts have been established, have the authority to take the lead in mediating the civil component. The mediation process must be properly documented, and any agreements reached shall be publicly disclosed in accordance with the law and subject to social oversight. Once confirmed by judicial ruling, mediation agreements shall be enforceable, and their performance shall be linked to sentencing incentives. Additionally, alternative forms of liability such as community service, targeted rectification, and public interest donations should be actively developed to achieve flexibility in the means of assuming responsibility.

#### 4.2.2 Distinguishing standards of proof: from “conflation” to “stratified application”

The conflation of criminal and civil standards of proof stems from the failure to separate procedures, stratify standards, and distinguish reasoning. It is necessary to complete the substantive determination of civil public interest harm while ensuring the validity of criminal convictions.

Procedural separation comes first. Experience with collective redress reform in Europe shows that public law regulation and private law remedies can each maintain their own distinct logic of proof within their respective legal systems. The key lies in assigning these two functions to different procedural entities, each retaining its own independent evidentiary review standards (29). In China’s criminal incidental civil public interest litigation in food safety, however, the procuratorial authority simultaneously performs the dual roles of criminal prosecutor and civil public interest representative, with both functions sharing a single procedural space. The conflation of standards of proof is thereby difficult to avoid. Therefore, a separate review stage for civil public interest litigation should be established within court proceedings to centrally address civil matters such as the scope of harm and the calculation of damages. This stage should allow the procuratorial authorities to present specialized materials such as expert opinions and audit reports, and to introduce expert assistants to appear in court for cross-examination, thereby providing an independent procedural space for the complex proof of civil harm (57).

The second is stratification of standards. For civil matters requiring proof, such as the facts of the harm and the amount involved, the more flexible standard of proof—“preponderance of the evidence”—should be explicitly applied (58). The court may consider materials such as scientific extrapolations based on the scale of violations, risk assessment reports from authoritative institutions, and sampling statistics as references for judicial discretion, while focusing on reviewing their scientific validity, relevance, and overall probative value in accordance with evidentiary rules.

The third is the separation of reasoning. Judgments must provide separate reasoning for the admissibility and use of evidence in the criminal and civil parts. Regarding the calculation of civil damages, if matters involve presumptions, expert opinions, or discretionary factors, the formation of judicial reasoning must be clearly articulated. This approach is not only the ultimate manifestation of differentiated evidentiary rules but also an essential requirement for using judicial transparency to enhance the quality of review and improve the credibility of adjudication.

#### 4.2.3 Standardizing punitive damages: from “unchecked discretion” to “unified rules”

To overcome the institutional challenges surrounding punitive damages, improvements are needed in the following three areas.

First, the legal basis for such claims should be clarified through legislation. In the future, provisions should be added to the Public Interest Litigation Law of the People’s Procuratorate to explicitly authorize procuratorial authorities to independently seek punitive damages when initiating criminal incidental civil public interest litigation in areas such as food safety. This measure aims to establish an independent right to claim “public interest penalties,” thereby fundamentally distinguishing it from private interest compensation intended to compensate for individual losses. This establishes the procuratorial authorities’ legal standing as representatives of the public interest in exercising this right (12, 13).

Second, a refined system of discretionary rules should be established. To achieve optimal deterrence with punitive damages, the multiplier must be inversely proportional to the probability of detecting the violation (59). When discretionary constraints are absent, the dispersion of award amounts increases sharply, and adjudicators may be unconsciously influenced by factors unrelated to the substance of the case, causing discretionary outcomes to deviate from normative goals (60). The extreme polarization and high dispersion of compensation amounts in the Chinese sample data resonate with these findings across different legal systems. Moreover, the triple factors of insufficient adversarial litigation structures, inadequate judicial review, and blocked channels for negotiation further amplify this universal challenge within the local context. Refining discretionary rules is therefore particularly urgent, and the core objective lies in establishing clear normative constraints in the following three areas.

1. Unifying the calculation base and setting a maximum limit. Specifically, the total amount of punitive damages for the entire case should be capped at the total price paid by end consumers as verified through evidence (61). Within this total, the respective shares of each defendant shall be determined comprehensively based on factors such as their subjective fault, the proportion of causation, and actual gains. Where joint intent exists, joint and several liability may be imposed, but the proportion of internal recourse must be clearly specified. Consideration of consumer fault should be exercised with extreme caution, and may only serve as an exceptional discretionary factor when the consumer’s own conduct is seriously unlawful and has no direct connection to the food safety risk. Professional claims involving “knowing buying” do not affect the determination of the operator’s illegality or the application of punitive damages. In addition, when the sales amount is truly difficult to ascertain, reasonable substitutes such as purchase cost, illegal gains, or fair market value may be used as the base, provided that the necessity of such substitution and the reasonableness of the calculation are fully substantiated in the judgment.
2. Adopting a tiered discretionary model. The wide variation in case circumstances requires that adjudicators retain the flexibility to adjust compensation amounts based on the specific facts; yet unchecked discretion inevitably leads to unpredictable outcomes. The key to resolving this tension lies in subjecting the exercise of discretion to a framework of accountability that is reasoned and reviewable. The trial court’s factual determinations should be subject to review and correction by the appellate court; each exercise of discretion must carry a duty of adequate reasoning and remain under the potential supervision of a higher court (62). Based on this, the practice of mechanically applying a fixed multiplier should be abandoned. Instead, a basic floating range of one to ten times may be established, accompanied by a tiered system for evaluating circumstances. In exercising discretion, courts should comprehensively consider key factors, including the subjective malice of the act, the scope of harm, whether the act targeted vulnerable groups, and whether actual health damage was caused. Cases should be classified into light, moderate, and severe tiers, ensuring that the multiplier of punitive damages corresponds substantively to the gravity of the violation.

The tiered model may be structured as follows:

- Basic Tier (1 to 3 times): Applicable to cases involving relatively minor subjective fault (such as negligence), first-time or isolated offenses, a short duration of the violation, and no verifiable health damage caused.
- Medium Tier (4 to 7 times): Applicable to cases where the offender acted with “knowing” intent, the scope of sales was relatively broad, the duration was relatively long, the risks associated with the food itself were clear, or the violation has caused minor health impacts on consumers.
- High Tier (8 to 10 times): Applicable to repeat or recidivist offenses; cases where the primary target consists of vulnerable groups such as pregnant women and infants; cases involving the illegal addition of highly toxic substances; or cases where particularly egregious circumstances—such as causing serious bodily harm to consumers—have resulted in extremely negative social impact.

This tiered structure draws on the concept of graduated penalties in administrative sanctions and criminal sentencing within the food safety sector, and aligns with the range of punitive damages specified in Article 55 of the *Consumer Rights and Interests Protection Law.* In addition, to ensure the reasonableness of the total amount and avoid judgments that are excessively high and difficult to enforce, consideration may be given to setting a limit on the total amount of punitive damages—in principle, not exceeding a specified multiple (e.g., three to five times) of the criminal fine—or establishing an absolute monetary cap based on regional economic conditions. This approach ensures a balance between the deterrent effect of the judgment and its practical enforceability. Proportionality between punitive damages and the gravity of the violation is a fundamental requirement of law and economics (59). *The EU Representative Actions Directive* likewise emphasizes that redress measures must not exceed what is necessary to compensate for the actual harm, and establishes the principle of proportionality as the fundamental guideline governing the relationship between the total amount of punitive damages and criminal fines—a position consistent with this logic.

1. Clarifying the principles governing the coordination of liability. The exercise of discretion shall be subject to the following principles: (i) the principle of separation of criminal and civil liability and overall balance, meaning that punitive damages and criminal fines are distinct in nature and function and shall not be offset against each other (63); however, when determining the multiplier, the amount of fines already imposed may be considered as a factor in the overall balance of liability (21); (ii) the principle of holistic evaluation of the entire supply chain, requiring that the production and sales chain be considered as a whole to avoid fragmentation of liability; (iii) the principle of prioritizing the return of illegal gains, which requires that all unlawful gains be ordered to be returned under Article 64 of the Criminal Code—such recovery serves as an important reference for determining internal allocation of punitive damages; and (iv) the principle of full judicial reasoning, which requires that judgments provide a detailed explanation of the determination of the amount, the allocation of liability, and the choice of multiplier, thereby constraining discretionary power through an open and transparent process and stabilizing expectations regarding judicial rulings.

Third, establish a priority order for enforcement and a mechanism for the dedicated management of funds. To ensure the practical effectiveness of “public interest penalties,” strict safeguards must be established at both the property enforcement and fund utilization levels. At the enforcement order level, the priority of civil liability (punitive damages) must be established. Judgments must explicitly state that, in cases where both criminal fines and punitive damages are imposed, punitive damages shall be satisfied first if the judgment debtor’s assets are insufficient to cover all debts. Concurrently, courts should establish a coordination mechanism between criminal and civil enforcement; when transferring cases for enforcement, they must clearly mark them as “priority for compensation in incidental civil public interest litigation,” ensuring strict adherence to this priority order during asset distribution. At the fund management level, a specialized and transparent management mechanism must be established. The proceeds of public interest punitive damages are distinct both from private interest punitive damages and from criminal fines. They are essentially intended to safeguard the diffuse interests of an unspecified group of consumers and represent the pricing of systemic risk. Therefore, they should not—and cannot—be simplistically classified as criminal fines or administrative penalties in the general sense and remitted to the state treasury (64). Instead, they should be collected and enforced strictly through judicial channels. Judgment documents should explicitly designate the compensation as “public interest special funds” and direct its transfer to a consumer rights protection public interest fund or a dedicated escrow account, thereby achieving a complete separation from forfeited revenues.

This direction is consistent with the emerging trend among Chinese judges: even in the absence of clear institutional norms, the practice of transferring punitive damages to specially managed accounts has become increasingly common in judicial practice (25). A functional reference is provided by the *cy près* doctrine in U.S. consumer class actions: when compensation cannot be distributed individually because the injured consumers are difficult to identify, courts may direct the remaining funds to the public interest use most closely related to the harmed interests, thereby preventing the legitimacy of collective redress from being undermined by unclear fund destinations (28). However, the practice of the *cy près* doctrine shows that the effective operation of a special fund cannot rely solely on case-by-case judicial discretion; it also requires institutional safeguards such as prior review of the eligibility of the recipient institution, mandatory disclosure of conflicts of interest, and ongoing supervision of fund use (65, 66).

In terms of specific institutional design, experience from other jurisdictions can be drawn upon. The U.S. Consumer Financial Protection Bureau (CFPB) has statutorily established a civil penalty fund, which is administered by a manager who distributes the funds to injured consumers according to a statutory order of priority; the distribution plan must be reported periodically and subject to public oversight^21^. *EU Directive 2024/1260*^22^ requires member states to take into account victims’ compensation claims in asset recovery and confiscation proceedings, ensuring that confiscated assets are preferentially used to compensate victims. Some civil law countries have experimented with a governance model of a fund committee involving courts, administrative authorities, and consumer associations, using a multi-party structure to respond to the risk of a loss of public confidence. Drawing on these experiences, a special fund combined with multi-party oversight is the institutional baseline for severing the mixing of funds with public finances and rebuilding a sense of public interest ownership.

With regard to the managing entity, based on considerations of neutrality and professionalism, priority may be given to provincial market regulatory authorities or consumer associations (14). The use of funds must follow a statutory order: first, to compensate consumers for actual losses that cannot otherwise be recovered; second, to cover necessary litigation costs; and the remainder shall be exclusively used for public interest initiatives such as food safety risk monitoring and public legal education (67). The managing entity must fulfill mandatory information disclosure obligations, publish audited annual financial reports, and voluntarily submit to public oversight.

## 5 Conclusion

This study has several limitations that warrant clarification. First, the sample is restricted to two publicly accessible judicial databases, China Judgments Online and the Peking University Law Database. Unpublished judicial documents (including cases resolved through non-public mediation or settlement) and judgments that have been withdrawn from public access cannot be included in the statistics. This may lead to a systematic underestimation of certain case types, particularly those where public interest repair was achieved outside the formal trial process.

Second, the coding framework can only capture information recorded in the judicial documents. Matters not mentioned in the documents—such as the actual enforcement rate of punitive damages, the specific allocation and use of the awarded funds after judgment, and the prosecutorial authorities’ discretionary activities before formal filing—are naturally beyond the reach of the coding tool.

Third, the geographical distribution of cases is significantly uneven, with some provinces accounting for a disproportionately high share of the sample. Consequently, regional variations in judicial practice may not be fully reflected in the overall statistical results. The findings have stronger explanatory power for provinces with a high number of cases and are relatively limited for provinces with sparse case volumes.

Fourth, the sample period covers the years 2021 to 2025, during which several relevant legal instruments were newly enacted or revised, and the Public Interest Litigation Law of the People’s Procuratorate is still under deliberation. The institutional environment remains in a state of flux. Future longitudinal research is needed to assess whether the structural dilemmas identified in this study persist, intensify, or dissipate as the legislative framework matures.

These limitations notwithstanding, this study provides an empirical examination of criminal incidental civil public interest litigation in food safety, based on an analysis of 677 judicial documents. The findings reveal systemic obstacles at every stage: an ambiguous definition of “social public interest,” rigid adherence to the “criminal proceedings first” order, conflation of criminal and civil standards of proof, unchecked discretion in punitive damages, and disparities in enforcement between fines and compensation funds.

These obstacles are not merely technical legal problems; they reflect deeper structural tensions between the logics of criminal and civil law within China’s evolving judicial governance system. The disproportionate emphasis on criminal procedure over civil remediation, the formalistic pre-litigation announcement, and the unbalanced ratio of punitive damages to criminal fines all point to a governance model that remains heavily criminal-centric. Addressing these tensions requires a fundamental shift from a rigid, sequential framework toward a flexible, remediation-oriented approach.

The policy implications are clear. China should establish a clear statutory standard for identifying “social public interest” in food safety cases, introduce a flexible trial model that allows early resolution of civil claims, clarify the respective standards of proof for criminal and civil components, and develop unified guidelines for punitive damages—including calculation bases, discretionary multipliers, and enforcement priorities. A transparent mechanism for managing punitive damages should also be established to ensure they directly serve the public interest in remediation.

Ultimately, this study offers a rule-of-law solution for food safety governance that integrates Chinese characteristics with universal values, presenting a China-inspired model to global legal discourse on public interest litigation and judicial governance.

Future research should expand the sample to include unpublished mediation documents and conduct comparative studies across provinces to assess regional variations. Longitudinal studies tracking the impact of the forthcoming Public Interest Litigation Law would also be valuable for understanding how institutional reforms translate into improved food safety outcomes.

## Data Availability

The original contributions presented in the study are included in the article/Supplementary material, further inquiries can be directed to the corresponding author.

## Footnotes

1 In 2017, the Standing Committee of the National People’s Congress adopted amendments to the *Civil Procedure Law and the Administrative Procedure Law*, formally establishing at the legislative level the standing and institutional framework for procuratorial organs to initiate public interest litigation, thereby achieving a profound transition from political decision-making to a legal system. In 2018, the Supreme People’s Court and the Supreme People’s Procuratorate jointly issued the “*Interpretation on Several Issues Concerning the Application of Law in Public Interest Litigation Cases*,” which, for the first time, clarified the rules for hearing criminal incidental civil public interest litigation, marking the entry of this system into a phase of standardized operation. Article 97 of the “*Rules on the Handling of Public Interest Litigation Cases by the People’s Procuratorates*,” revised in 2020, stipulates that the People’s Procuratorate may initiate criminal incidental civil public interest litigation against unlawful acts in the field of food and drug safety that infringe upon the lawful rights and interests of numerous consumers, making this one of the key forms of procuratorial public interest litigation in practice.

2 Article 58 of the *Civil Procedure Law and relevant provisions such as the Interpretation on Several Issues Concerning the Application of Law in Public Interest Litigation Cases* merely refer in principle to “harm to social public interest,” but do not provide specific rules regarding its connotation, extension, criteria for determination, or methods for quantifying harm in the context of food safety and other fields. Upon examination, the Legislative Affairs Commission of the Standing Committee of the National People’s Congress (the Commission), as the legislative body, has not issued any generally binding interpretation on the specific application of “social public interest” in consumer public interest litigation, as reflected in its publicly available legislative interpretations, responses to inquiries, or filing review cases. In practice, the concretization of this concept relies primarily on case-by-case discretion by courts at various levels.

3 *Civil Procedure Law of the People’s Republic of China*, Article 58 provides: “For acts that pollute the environment, infringe upon the lawful rights and interests of numerous consumers, or otherwise harm the social public interest, the authorities and relevant organizations as prescribed by law may bring a lawsuit before a people’s court.”

4 See Criminal Incidental Civil Judgment of the Hetang District People’s Court of Zhuzhou City, Hunan Province (2021) Xiang 0202 Criminal First Instance No. 324.

5 See Criminal Incidental Civil Judgment of the Bayuquan District People’s Court of Yingkou City, Liaoning Province (2021) Liao 0804 Criminal First Instance No. 16.

6 See Criminal Incidental Civil Judgment of the Huangzhong County People’s Court of Qinghai Province (2024) Qing 0122 Criminal First Instance No. 401.

7 See Criminal Incidental Civil Judgment of the Shuangjiang Lahu, Wa, Blang and Dai Autonomous County People’s Court of Yunnan Province (2025) Yun 0925 Criminal First Instance No. 110.

8 See Criminal Incidental Civil Judgment of the Qibin District People’s Court of Hebi City, Henan Province (2021) Yu 0611 Criminal First Instance No. 294.

9 See *Lyu Moumou and Pang Moumou Selling Toxic or Harmful Food* case, Criminal Incidental Civil Judgment of the Laiwu District People’s Court of Jinan City, Shandong Province (2020) Lu 0116 Criminal First Instance No. 561; and *Ma Jialiang Selling Toxic or Harmful Food* case, Criminal Incidental Civil Judgment of the Jinxiang County People’s Court of Shandong Province (2022) Lu 0828 Criminal First Instance No. 141.

10 See Criminal Incidental Civil Judgment of the Dongcheng District People’s Court of Beijing Municipality (2023) Jing 0101 Criminal First Instance No. 515.

11 See Criminal Incidental Civil Judgment of the Hechuan District People’s Court of Chongqing Municipality (2020) Yu 0117 Criminal First Instance No. 610; Criminal Incidental Civil Judgment of the Second People’s Court of Zhongshan City, Guangdong Province (2024) Yue 2072 Criminal First Instance No. 554.

12 See Criminal Incidental Civil Judgment of the Huaibei City Intermediate People’s Court of Anhui Province (2024) Wan 06 Criminal Final No. 137; Criminal Incidental Civil Judgment of the Baise City Intermediate People’s Court of Guangxi Zhuang Autonomous Region (2024) Gui 10 Criminal Final No. 245.

13 For example, in the case of *Li Yuanwang Selling Food That Did Not Meet Safety Standards* (see Criminal Incidental Civil Judgment of the Shuangjiang Lahu, Wa, Blang and Dai Autonomous County People’s Court of Yunnan Province (2025) Yun 0925 Criminal First Instance No. 110) and the case of *Wei Moujia Producing Toxic or Harmful Food* (see Criminal Incidental Civil Judgment of the Helan County People’s Court of Ningxia Hui Autonomous Region (2025) Ning 0122 Criminal First Instance No. 118), the terms of the mediation agreements were to pay compensation equal to once the sales amount (2,700 yuan) and twice the sales amount (14,812 yuan), respectively (the latter also requiring a public apology in a newspaper). The terms of the mediation in both cases were identical to the claims stated in the indictments, and there is no record of substantive negotiation regarding the standards of compensation or methods of remediation.

14 For example, in the case of *Liu Moujia A Selling Toxic or Harmful Food* (see Criminal Incidental Civil Judgment of the Ruzhou City People’s Court of Henan Province (2021) Yu 0482 Criminal First Instance No. 457) and the case of *Yang Moumou Selling Toxic or Harmful Food* (see Criminal Incidental Civil Judgment of the Ruzhou City People’s Court of Henan Province (2021) Yu 0482 Criminal First Instance No. 479), the procuratorial authorities filed motions to dismiss the cases after the defendants’ relatives voluntarily paid punitive damages (1,000 yuan and 8,000 yuan, respectively). The dismissals were entirely contingent upon the fulfillment of the monetary obligations specified in the indictments, reflecting the most conservative approach to debt recovery.

15 Typical examples include the *Wang Moumou and Yin* case, where civil liability was borne entirely by the principal offender, Wang Moumou, while the accessory, Yin, was not held liable; and the *Yao Moumou and Bai Moumou* case, where the two defendants were found to have committed a joint tort, yet each was held liable only in proportion to their respective sales amounts, without establishing joint and several liability or internal allocation. See Criminal Incidental Civil Judgment of the Ar Horqin Banner People’s Court of Inner Mongolia Autonomous Region (2024) Nei 0421 Criminal First Instance No. 72; Criminal Incidental Civil Judgment of the Lishi District People’s Court of Lüliang City, Shanxi Province (2025) Jin 1,102 Criminal First Instance No. 49.

16 Practices vary in actual cases: in the *Li and Xiao* case, among others, the liability of the upstream seller Li was limited to the scope of “part of the downstream sales amount”; whereas in the *Zhu Moujia and Hong* case, the liability of the upstream seller Hong was limited to a multiple of Zhu’s “purchase price.” In both approaches to apportioning liability, the choice of calculation benchmark lacks sufficient legal justification. See Criminal Incidental Civil Judgment of the Huai’an Economic and Technological Development Zone People’s Court of Jiangsu Province (2024) Su 0891 Criminal First Instance No. 57; Criminal Incidental Civil Judgment of the Sixian County People’s Court of Anhui Province (2025) Wan 1,324 Criminal First Instance No. 324.

17 In the *Wang Liqin* case, the court exempted the defendant from punitive damages on the grounds that the consumer had committed the illegal act of consuming a protected animal, provided that liability was established; whereas in the *Zhang et al.* case, the court dismissed the plaintiffs’ claims on the grounds of lack of standing, ruling that they were professional claimants who engaged in “knowing buying” rather than consumers. See Criminal Incidental Civil Judgment of the Wujin District People’s Court of Changzhou City, Jiangsu Province (2021) Su 0412 Criminal First Instance No. 34; Criminal Incidental Civil Judgment of the Linze County People’s Court of Gansu Province (2023) Gan 0723 Criminal First Instance No. 5.

18 See, e.g., the *Zhu Moujia and Hong* case, Criminal Incidental Civil Judgment of the Sixian County People’s Court of Anhui Province (2025) Wan 1,324 Criminal First Instance No. 324; and the *Ma Mouming* case, Criminal Incidental Civil Judgment of the Xiushan Tujia and Miao Autonomous County People’s Court of Chongqing Municipality (2024) Yu 0241 Criminal First Instance No. 141.

19 For example, in the *Wang Moumou and Yin* case, the court, in response to the defense counsel’s objection based on the principle of *ne bis in idem* (no double jeopardy), explicitly stated that criminal fines and punitive damages “differ in nature”; in the *Wen Yaxin* case, the court directly ruled that “criminal fines constitute criminal liability, while punitive damages constitute civil liability, and there is no legal basis for offsetting the two.” See Criminal Incidental Civil Judgment of the Ar Horqin Banner People’s Court of Inner Mongolia Autonomous Region (2024) Nei 0421 Criminal First Instance No. 72; Criminal Incidental Civil Judgment of the Baoting Li and Miao Autonomous County People’s Court of Hainan Province (2023) Qiong 9,029 Criminal First Instance No. 15.

20 Wal-Mart Stores, *Inc. v. Dukes*, 564 U.S. 338, 349 (2011).

21 Consumer Financial Civil Penalty Fund Rule, 12\u00B0C.F.R. § 1075.104 (2013).

22 Directive (EU) 2024/1260 of the European Parliament and of the Council of 24 April 2024 on asset recovery and confiscation [2024] OJ L169/1.
